# Geographic range size and extinction risk assessment in nomadic species

**DOI:** 10.1111/cobi.12440

**Published:** 2015-01-09

**Authors:** Claire A Runge, Ayesha Tulloch, Edd Hammill, Hugh P Possingham, Richard A Fuller

**Affiliations:** * School of Biological Sciences, University of QueenslandBrisbane, QLD, 4072, Australia; † School of Geography, Planning and Environmental Management, University of QueenslandBrisbane, QLD, 4072, Australia; ‡ School of the Environment, University of TechnologySydney, NSW, 2007, Australia; § Imperial College London, Department of Life SciencesSilwood Park, Ascot SL5 7PY, Berkshire, England, United Kingdom

**Keywords:** arid zone, conservation priority setting, geographic range size, IUCN Red List, migration, nomadism, species distribution modeling, threatened species, especie amenazada, establecimiento de prioridades de conservación, lista roja de la UICN, migración, modelado de distribución de especies, nomadismo, tamaño de extensión geográfica, zona árida

## Abstract

Geographic range size is often conceptualized as a fixed attribute of a species and treated as such for the purposes of quantification of extinction risk; species occupying smaller geographic ranges are assumed to have a higher risk of extinction, all else being equal. However many species are mobile, and their movements range from relatively predictable to-and-fro migrations to complex irregular movements shown by nomadic species. These movements can lead to substantial temporary expansion and contraction of geographic ranges, potentially to levels which may pose an extinction risk. By linking occurrence data with environmental conditions at the time of observations of nomadic species, we modeled the dynamic distributions of 43 arid-zone nomadic bird species across the Australian continent for each month over 11 years and calculated minimum range size and extent of fluctuation in geographic range size from these models. There was enormous variability in predicted spatial distribution over time; 10 species varied in estimated geographic range size by more than an order of magnitude, and 2 species varied by >2 orders of magnitude. During times of poor environmental conditions, several species not currently classified as globally threatened contracted their ranges to very small areas, despite their normally large geographic range size. This finding raises questions about the adequacy of conventional assessments of extinction risk based on static geographic range size (e.g., IUCN Red Listing). Climate change is predicted to affect the pattern of resource fluctuations across much of the southern hemisphere, where nomadism is the dominant form of animal movement, so it is critical we begin to understand the consequences of this for accurate threat assessment of nomadic species. Our approach provides a tool for discovering spatial dynamics in highly mobile species and can be used to unlock valuable information for improved extinction risk assessment and conservation planning.

Tamaño de Extensión Geográfica y Evaluación de Riesgo de Extinción en Especies Nómadas

**Resumen:**

El tamaño de extensión geográfica se conceptualiza frecuentemente como un atributo fijo de las especies y se trata como tal para los propósitos de cuantificación de riesgo de extinción; se asume que las especies que ocupan extensiones geográficas más pequeñas tienen un riesgo de extinción más alto, cuando todo lo demás es igual. Sin embargo, muchas especies son móviles y sus movimientos varían desde migraciones de ida y vuelta relativamente predecibles hasta movimientos irregulares complejos, como los que muestran las especies nómadas. Estos movimientos pueden llevar a expansiones sustanciales temporales y a una reducción de las extensiones geográficas, todo esto con el potencial de llegar a niveles que pueden presentar un riesgo de extinción. Al enlazar los datos de presencia con las condiciones ambientales al momento de la observación de las especies nómadas pudimos modelar las distribuciones dinámicas de 43 especies de aves de zonas áridas a lo largo de la isla de Australia durante cada mes a lo largo de once años y calculamos el tamaño de extensión mínima y el alcance de las fluctuaciones en el tamaño de extensión geográfica a partir de estos modelos. Hubo una enorme variabilidad en la distribución espacial pronosticada a lo largo del tiempo: diez especies variaron en el tamaño de extensión geográfica por más de una orden de magnitud y dos especies variaron por más de dos órdenes de magnitud. Durante situaciones de condiciones ambientales pobres, varias especies que actualmente no se encuentran clasificadas como amenazadas a nivel global redujeron sus extensiones a áreas muy pequeñas, esto a pesar de su gran tamaño de extensión geográfica normal. Este hallazgo genera preguntas sobre lo idóneo de las evaluaciones convencionales del riesgo de extinción con base en el tamaño estático de extensión geográfica (p. ej.: la Lista Roja de la UICN). Se pronostica que el cambio climático afectará los patrones de las fluctuaciones de recursos en casi todo el hemisferio sur, donde el nomadismo es la forma dominante de movimiento de animales, así que es crítico que comencemos a entender las consecuencias de esto para tener una evaluación certera del riesgo de extinción de especies nómadas. Nuestra estrategia proporciona una herramienta para descubrir las dinámicas espaciales de especies con movilidad alta y puede usarse para liberar información valiosa para una mejor evaluación de riesgo de extinción y planeación de la conservación.

## Introduction

Extinction risk estimates provide one of the foundations for prioritizing conservation actions (Joseph et al. 2009), but their usefulness is hindered by a lack of accurate distribution and abundance metrics for many species. Measures of geographic range size can be used as surrogates for population decline and extinction risk (Purvis et al. 2000); geographic range size consistently emerges as a key correlate of extinction risk in mammals, amphibians, and birds (Cardillo et al. 2008; Sodhi et al. 2008; Lee & Jetz 2011). Several different measures of geographic range size exist (Gaston & Fuller 2009). Estimations of extinction risk are typically calculated using static metrics such as extent of occurrence (EOO) or area of occupancy (AOO), which are based on a conceptualization of geographic range size as a fixed attribute of a species. EOO is a measure of the degree to which a species’ distribution, and hence its vulnerability to threats, is spread across geographic space, and AOO is a measure of the area actually occupied by the species. With these metrics, species with smaller extents or areas are assumed to be more threatened (Gaston & Fuller 2009; IUCN 2014). However, when a species is nomadic within its overall distribution, estimates of EOO or AOO based on pooling observations across time will often be larger than the geographic range size at any one point in time. This could lead to an erroneous conclusion that a nomadic species is safe from extinction when it is not. We examined the temporal variability in the AOO of nomadic species and explored the consequences of such dynamism for extinction risk assessments.

Across much of the southern hemisphere, animal movement patterns are dynamic and irregular, and many bird species display some form of irregular movement such as nomadism (Chan 2001; Dean 2004). Nomads move in complex patterns, often associated with highly fluctuating resources, for example, seasonal fruiting or resource booms associated with irregular desert rainfall (Berthold 2001; Dean 2004; Cox 2010). Movement strategies may be adjusted dynamically according to the prevailing conditions at each time and place (Andersson 1980; Webb et al. 2014). Much of the information on nomadic movements in individual species is anecdotal or qualitative, likely as a result of the difficulties in monitoring and tracking such highly dynamic species (Marchant & Higgins 1990). As a consequence, the responses by nomads to fluctuations in environmental conditions remain poorly understood (Bennetts & Kitchens 2000; Dean & Milton 2001). Without this information, it is challenging to estimate their extinction risk.

Almost 50% (2072 of 4440 species) of threatened species are listed as threatened on the basis of geographic range size criteria and meet subcriteria on population trends, fragmentation, and fluctuations (Gaston & Fuller 2009). However, any measure of geographic range size for nomadic species that pools distributional data across time represents a maximum that is an upper bound on a distribution. At certain points in time a species’ distribution might contract to localized resource patches, and the species will occupy only a very small part of its maximum distribution. Moreover, many nomadic species move large distances across inaccessible environments that are poorly surveyed, leading to large gaps in our knowledge of their distributions (Szabo et al. 2007; Tulloch et al. 2013). These gaps make it difficult to determine from distributional data alone whether a species is in a true contraction and missing from much of the landscape or whether surveys have not adequately covered its whole distribution.

The consequences of range fluctuations on species’ persistence are partially captured by existing extinction risk assessment frameworks; extreme fluctuation is an assessable subcriterion under criteria B and C2 of the International Union for Conservation of Nature (IUCN) Red List (IUCN 2014). However, IUCN red listing under extreme fluctuation is only triggered once a species drops below population size or geographic range size thresholds. Lack of theoretical and empirical testing leaves the relationship between fluctuating range sizes and extinction risk unclear, though there is evidence for higher extinction risk in both species with fluctuating population sizes (Pimm et al. 1988; Hung et al. 2014) and those experiencing temporary range contraction (Newton 2004); this forms the basis for IUCN Red List criterion B (IUCN 2014). However, actual relationships are likely to be species- and threat-specific, depending on the nature of threats and the impact those threats have on density–occupancy relationships in the target species (Gaston 2003).

Several previous studies have used modeling to identify fluctuating species distributions (Reside et al. 2010; Bateman et al. 2012; Sardà-Palomera et al. 2012), though the extent of geographic range size fluctuations in vertebrates remains poorly known. We determined temporal variability in the geographic range size (i.e., AOO) and therefore extinction risk for a suite of Australian nomadic birds. We compared time-sliced estimates of AOO (i.e., monthly estimates based on modeled distribution maps) against more traditional estimates of AOO based on occurrences of taxa pooled across time. We used the results of our models to provide guidelines for incorporating range size variability into existing extinction risk assessments.

## Methods

### Case Study Area and Species

We used a suite of Australian arid-zone nomadic birds as a case study. Occupying over 6.2 million km^2^, the Australian arid and semiarid zones are associated with irregular fluctuations in resources predominantly driven by rainfall. Complex patterns of rainfall drive movement in many species of birds, mammals, and invertebrates (Keast 1959; Dean 2004; Letnic & Dickman 2006). Resource fluctuations comprise annual seasonality overlain onto longer scale and less predictable boom-and-bust cycles in resources (Meyers et al. 2007; Risbey et al. 2009). Nomadic species in Australia face a suite of threats, including habitat loss through degradation and human encroachment, climate change, and pressure from introduced species (Reid & Fleming 1992; Cleugh et al. 2011; Ford 2011; Garnett et al. 2011).

We selected 43 arid-zone bird species described as nomadic or possibly nomadic (Marchant & Higgins 1990; Ziembicki & Woinarski 2007; BirdLife International 2012). Bird occurrences were collated from 20 minute area searches of 2 ha plots conducted from June 2000 to March 2011 as part of the New Atlas of Australian Birds (for details see http://www.birdlife.org.au/projects/atlas-and-birdata). We excluded occurrences with no recorded coordinate system or where the spatial accuracy of the coordinate location was coarser than 500 m. The number of occurrences for each species ranged from 29 to 21,634 over the 11 years. We excluded occurrences outside Interim Biogeographic Regionalisation Areas (AGDoE 2004) that intersected Australian rangelands (ACRIS 2005) to limit model fitting to the arid and semiarid subpopulations of modeled species. We also excluded occurrences with missing environmental data (e.g., where cloud cover consistently disrupted satellite data). The study area was divided into gridded pixels of 0.05° for analysis.

### Species Distribution Models

We used the software Maxent v3.3.3 (Phillips et al. 2006) to predict the distribution of each species from the occurrence data sets. Maxent was run on an Ubuntu platform with samples-with-data inputs (Phillips et al. 2009). We accounted for coastal and spring bias in survey effort (Szabo et al. 2007) by drawing 10,000 background data points from a random sample of atlas surveys (Phillips et al. 2009).

We included 19 predictor variables in the models; twelve static variables (vegetation types), and 7 time-dependent variables calculated over the 3 months prior to the date of each record (maximum temperature, minimum temperature, maximum and normalized fractional photosynthetic vegetation (PV), maximum and normalized fractional nonphotosynthetic vegetation (NPV), Foley's drought index). For example, species occurrences for June 2000 were associated with environmental records aggregated over the months March 2000, April 2000, and May 2000. Short-term averages of weather data have been shown to predict nomadic species’ distributions more accurately than long-term climate averages (Reside et al. 2010). Time lags of 1, 2, 3, 4, 5, 6, and 12 months were tested, and 3 months emerged as the best predictor across the modeled species (but see Reside et al. 2010). All variables showed pairwise Pearson correlation coefficients below 0.7.

We calculated static vegetation variables by reclassifying the 31 National Vegetation Information System (NVIS)—Major Vegetation Groups Version 3.0 (AGDoE 2005) into 12 groups and calculating the proportion of each pixel covered by each vegetation group (Supporting Information). Fractional PV (vegetation greenness) and non-NPV (vegetation dryness) were calculated from the Guerschman FPV data set (Guerschman et al. 2009), which is based on remote-sensing data from the EO-1 Hyperion and MODIS satellites. We calculated maximum PV and NPV as the absolute maximum value over the 3-month window and normalized PV and NPV as that maximum divided by the long-term average for 2000 to 2011. We calculated 3-monthly maximum and minimum temperature from interpolated daily temperatures accessed through SILO (Jeffrey et al. 2001). Foley's drought index was used to reflect rainfall scarcity because rainfall is interpolated across large distances in the study region (Fensham et al. 2009).

We created one species distribution model for each species from all records spanning June 2000 to March 2011. We assumed that the modeled species respond consistently to environmental drivers across their range. For each species, we projected the model onto the environmental variables corresponding to each month from that period to create monthly time-sliced distributions (130 projections per species). We validated models with a combination of null model testing, comparison with published distributions, and expert evaluation based on known ecology. Null models were created by selecting 100 random subsets from all survey data; the number of records corresponded to the number of records used to model each species. All species models had greater predictive power than null models run with the same parameters (*z* test; probability that observed model area under the curve of receiver operating characteristic [AUC] falls within the range expected from the null model *p* < 0.00001 for all species [Raes & ter Steege 2007]). We rejected one species on the basis of a visual assessment of the resulting distribution maps (the cryptic Chestnut-backed Quail-thrush [*Cinclosoma castanotum*]), which showed low probability of environmental suitability in some areas of known habitat. This species is a cryptic ground-dwelling bird with a call above the hearing range of many observers, likely making this species’ observations heavily affected by detectability bias.

We reclassified the Maxent logistic probability into predictions of absence and probability of presence using equal sensitivity and specificity threshold values (Liu et al. 2005). Each pixel above the threshold retained its logistic probability value of environmental suitability, whereas every pixel below the threshold was reclassified as zero suitability. We then clipped the time-sliced maps to exclude IBRA bioregions (AGDoE 2004) where the target species had not been detected in the 11 years. Due to the coarse spatial resolution of our distribution models, one pixel may contain multiple vegetation types, not all of which will be suitable for all species. Although a single pixel could be predicted as suitable, the entire area of that pixel (approximately 25 km^2^) is unlikely to be occupied. We therefore estimated geographic range size (i.e., AOO) at each point in time by multiplying the probability of environmental suitability of each pixel by the area (km^2^) of that pixel and then summing the values across all pixels in the time-sliced map. To derive an estimate of the pooled geographic distribution for each species, based on aggregated distribution across time (the kind of quantity typically used to estimate extinction risk for nomadic species), we calculated the maximum environmental suitability for each pixel across all periods, multiplied the suitability for each pixel by its area, and then summed the values across all pixels in the map.

### Extinction Risk

Minimum, maximum, and mean geographic range size calculated from the time-sliced range sizes are essentially akin to estimates of AOO (Gaston & Fuller 2009). We used linear models to analyze the relationship between the pooled geographic range size and the response variables of minimum, maximum, and mean range sizes estimated from our models. We calculated the magnitude of fluctuation as the ratio of maximum to minimum geographic range size and classified it as extreme fluctuation when that value exceeded 10 (IUCN 2014). Analyses were conducted using R version 2.15.1 (www.r-project.org) using the raster package (Hijmans et al. 2012).

## Results

**Figure 1:**
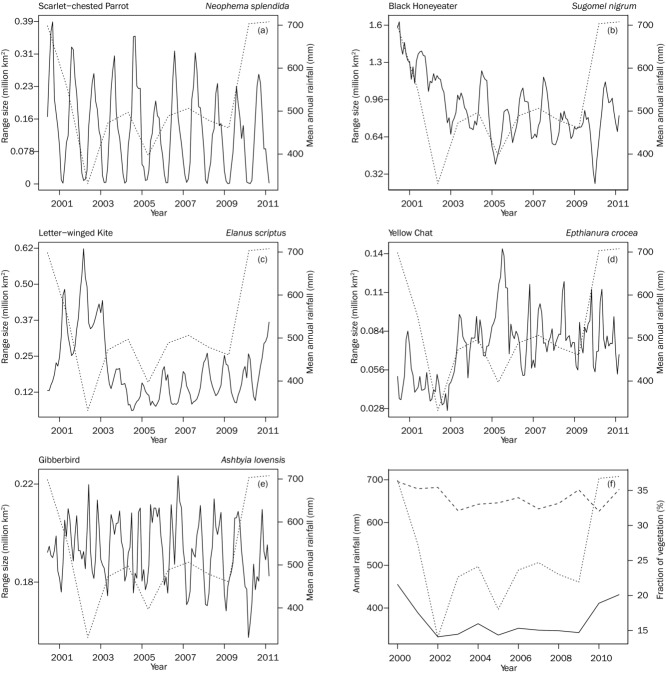
Examples of temporal dynamics in geographic range size for birds in arid Australia: (a) Scarlet-chested Parrot, (b) Black Honeyeater, (c) Letter-winged Kite, (d) Yellow Chat, (e) Gibberbird (dotted lines, mean annual rainfall for Australia for the period). (f) Mean annual rainfall (dotted line) relative to mean annual fraction of photosynthetic vegetation (solid line) and mean annual fraction of non-photosynthetic vegetation (dashed line) across Australia from 2000 to 2011.

The total area predicted as suitable for each species fluctuated across seasons and years with distinctly different patterns among species. The enormous variation in dynamics across species suggests that the models reflected the different relationship between each species and the environmental variables rather than the variation in particular environmental variables. Plots and animated maps of temporal range size dynamics are provided in Supporting Information. By way of example, the modeled range size for the Scarlet-chested Parrot (*Neophema splendida*) showed a strong degree of seasonal fluctuation with repeated seasonal minima in March (Fig.1a). This seasonal fluctuation was overlain with longer term fluctuation in both minima and maxima. Sixteen species (35%) showed such seasonal fluctuations.

Not all species showed extreme seasonal effects; 27 species (63%) exhibited some seasonal variation superimposed onto more complex dynamics. For instance, the Black Honeyeater (*Sugomel nigrum*) displayed slight seasonal variation but much stronger and more complex long-term effects (Fig.1b). At the beginning of the period, which corresponded to high rainfall across interior Australia (2000 to late 2002), the species was predicted to occupy a large area. Notably, the minima in these years exceeded the maxima of later years, and the distribution contracted to a low in January 2010.

Species showed mixed responses to landscape-wide dynamics in rainfall and drought. Letter-winged Kite (*Elanus scriptus*) ranges contracted dramatically corresponding to landscape-wide drought after 2003 and expanded to postdrought levels at the end of the time series (Fig.1c). These nocturnal raptors feed on rodents whose populations irrupt after high rainfall events such as those in 2000 to 2002 (Pavey et al. 2008). Recently there has been a spike in records corresponding with the latest rainfall event in 2009 to 2011 (Fig.1c & 1f) (Pavey & Nano 2013). Six other species showed a similar pattern (Black-shouldered Kite [*Elanus axillaris*]; Spotted Harrier [*Circus assimilis*]; Stubble Quail [*Coturnix pectoralis*]; Mistletoebird [*Dicaeum hirundinaceum*]; Black Falcon [*Falco subniger*]; Budgerigar [*Melopsittacus undulatus*]). An additional 7 species showed a weaker time-lagged contraction after 2003 with no recovery after 2009 (Grey Honeyeater [*Conopophila whitei*]; Ground Cuckooshrike [*Coracina maxima*]; Grey-headed Honeyeater [*Ptilotula keartlandi*]; Grey-fronted Honeyeater [*Ptilotula plumula*]; White-fronted Honeyeater [*Purnella albifrons*]; Black Honeyeater). Conversely, the habitat for 3 species expanded as the landscape dried out after 2003 (Fig.1d; Yellow Chat [*Epthianura crocea*]; Orange Chat [*Epthianura aurifrons*], and Chestnut-breasted Whiteface [*Aphelocephala pectoralis*]).

Interestingly, one species, the Gibberbird (*Ashbyia lovensis*), a species usually described in the literature as nomadic or locally nomadic (Marchant & Higgins 1990), displayed an approximately constant range size even though the location of these areas was dynamic (Fig.1e & Supporting Information).

**Figure 2:**
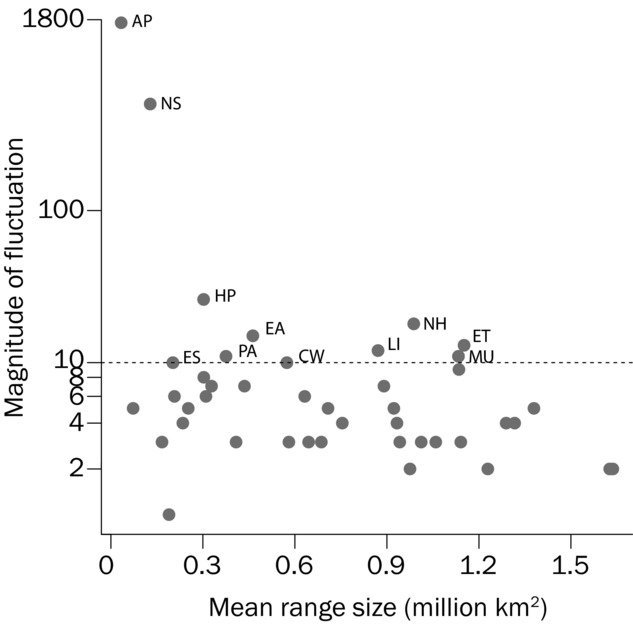
Mean modeled geographic range size relative to the magnitude of fluctuation in range size (maximum range size divided by minimum range size) for 43 nomadic species. Those species with fluctuations between minimum and maximum range size of more than one order of magnitude are labeled (AP, *Aphelocephala pectoralis*; NS, *Neophema splendida*; HP, *Heteromunia pectoralis*; NH, *Nymphicus hollandicus*; EA, *Elanus axillaris*; ES, *Elanus scriptus*; LI, *Lichmera indistincta*; ET, *Epthianura tricolor*; MU, *Melopsittacus undulatus*; PA, *Purnella albifrons*; CW, *Conopophila whitei*).

**Figure 3:**
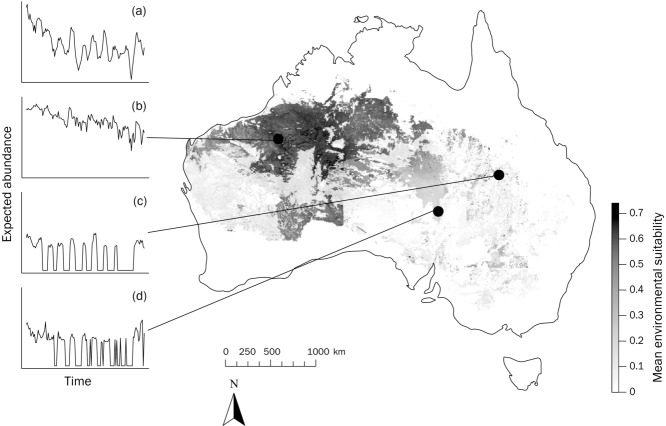
Theoretical outcome of monitoring abundance of Black Honeyeater across different geographic locations: (a) overall trend and (b) population dynamics at the core and (c-d) edges of the species’ overall range. A linear relationship between environmental suitability and abundance is assumed. Shading bar represents the mean probability that a pixel is environmentally suitable for the species.

Some species showed extreme fluctuations between the maximum and minimum range size (Fig.2), and the magnitude of these fluctuations increased as mean range size decreased. In part this is inevitable because fluctuation of the wider ranging species is limited by the size of the Australian continent. Of the 43 species, 11 showed extreme fluctuation (>1 order of magnitude) (Table1) as defined by IUCN Red List criterion B2cii (IUCN 2014). Trends in environmental suitability fluctuated markedly according to geographic location and position in the species’ range. In the case of the Black Honeyeater, sites in the core of the species range showed little variation in environmental suitability (Fig.3b) relative to sites at the margin of the species’ geographic distribution (Fig.3c & 3d).

**Table 1:** Range size and extinction risk metrics for 43 nomadic bird species

Common name	Scientific name	Pooled range size (km^2^)	Minimum range size (km^2^)	Magnitude of fluctuation in range size	Satisfies criterion B2 (range size < 2000 km^2^)	Satisfies subcriterion B2cii (extreme fluctuation)
Stubble Quail	*Coturnix pectoralis*	1,819,376	169,017	7		
Black-shouldered Kite	*Elanus axillaris*	2,645,411	113,305	15		yes
Letter-winged Kite	*Elanus scriptus*	719,691	60,454	10		yes
Spotted Harrier	*Circus assimilis*	3,559,606	583,026	4		
Australian Bustard	*Ardeotis australis*	3,135,949	1,123,919	2		
Common Bronzewing	*Phaps chalcoptera*	1,097,672	86,879	6		
Flock Bronzewing	*Phaps histrionica*	916,107	84,554	8		
Diamond Dove	*Geopelia cuneata*	2,731,995	220,878	9		
Grey Falcon	*Falco hypoleucos*	2,572,585	882,558	2		
Black Falcon	*Falco subniger*	2,675,534	537,230	3		
Major Mitchell's Cockatoo	*Lophochroa leadbeateri*	2,404,222	560,730	3		
Cockatiel	*Nymphicus hollandicus*	3,270,352	106,111	18		yes
Bourke's Parrot	*Neopsephotus bourkii*	1,657,523	746,496	2		
Scarlet-chested Parrot	*Neophema splendida*	496,793	776	502	yes	yes
Budgerigar	*Melopsittacus undulatus*	2,789,945	186,998	11		yes
Black Honeyeater	*Sugomel nigrum*	2,206,769	237,940	7		
Pied Honeyeater	*Certhionyx variegatus*	2,538,637	630,913	3		
Brown Honeyeater	*Lichmera indistincta*	2,571,125	138,958	12		yes
Painted Honeyeater	*Grantiella picta*	780,039	92,922	4		
Striped Honeyeater	*Plectorhyncha lanceolata*	659,307	82,817	5		
Gibberbird	*Ashbyia lovensis*	327,149	151,157	1		
Crimson Chat	*Epthianura tricolor*	2,611,986	157,107	13		yes
Orange Chat	*Epthianura aurifrons*	2,138,565	493,032	3		
Yellow Chat	*Epthianura crocea*	257,089	26,570	5		
White-fronted Chat	*Epthianura albifrons*	625,249	64,954	6		
Grey Honeyeater	*Conopophila whitei*	1,297,181	108,314	10		yes
Spiny-cheeked Honeyeater	*Acanthagenys rufogularis*	2,063,826	448,022	4		
White-fronted Honeyeater	*Purnella albifrons*	1,669,300	103,538	11		yes
Grey-headed Honeyeater	*Ptilotula keartlandi*	1,814,667	185,357	6		
Grey-fronted Honeyeater	*Ptilotula plumula*	2,210,412	255,598	5		
Striated Pardalote	*Pardalotus striatus*	1,161,005	219,578	3		
Western Gerygone	*Gerygone fusca*	2,271,607	384,749	4		
Chestnut-breasted Whiteface	*Aphelocephala pectoralis*	71,193	37	1720	yes	yes
Banded Whiteface	*Aphelocephala nigricincta*	1,446,464	336,688	3		
Ground Cuckooshrike	*Coracina maxima*	3,155,208	448,945	5		
Grey Fantail	*Rhipidura albiscapa*	436,107	86,055	3		
Little Crow	*Corvus bennetti*	2,508,774	1,111,711	2		
Jacky Winter	*Microeca fascinans*	1,564,910	326,901	3		
Red-capped Robin	*Petroica goodenovii*	2,829,147	562,818	4		
Mistletoebird	*Dicaeum hirundinaceum*	2,873,533	336,324	5		
Painted Finch	*Emblema pictum*	1,494,337	350,768	3		
Plum-headed Finch	*Neochmia modesta*	955,399	89,282	7		
Pictorella Mannikin	*Heteromunia pectoralis*	1,284,739	33,227	26		yes

**Figure 4:**
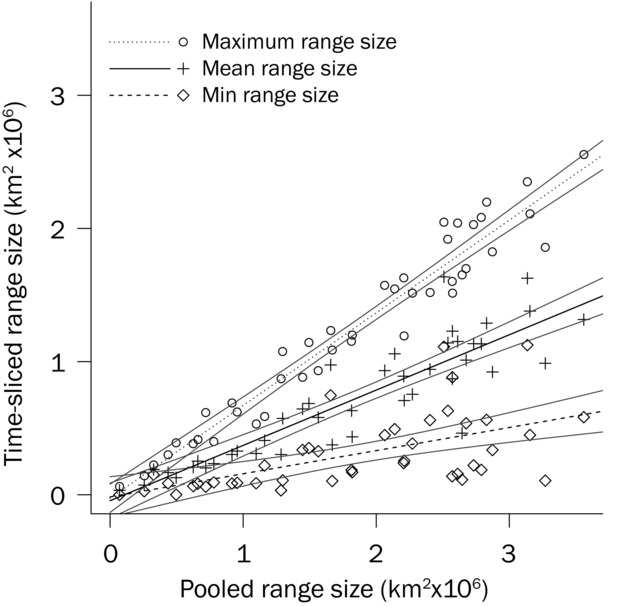
The relationship between pooled geographic range size and the time sliced (i.e., mapped dynamically across time) estimates of maximum (*y*∼0.70*x* − 2.6 × 10^4^, *p* < 0.001), mean (*y*∼0.40*x* − 4.4 × 10^4^, *p* < 0.001), and minimum (*y*∼0.17*x* − 1.6 × 10^4^, *p* < 0.001) range sizes. Bounding lines indicate 95% confidence intervals.

The slopes of linear models showed that pooled geographic range size exceeded the minimum geographic range size by 82.6% (95% CI 7.6), mean geographic range size by 58.5% (95% CI 6.6) and maximum geographic range size by 30.4% (95% CI 5.3) (Fig.4).

## Discussion

We conducted an empirical analysis of nomadic species dynamics, using time-sliced species distribution models linked to time-delayed local weather patterns. As expected, the area occupied was highly variable across time and the extent and pattern of fluctuation differed markedly among species. All species exhibited significant bottlenecks (i.e., points in time where the AOO of the species was very low). By exploring these bottlenecks using our estimates of minimum range size, we determined how many species met the classification thresholds for threat under IUCN guidelines. This approach can be applied with fewer data than quantitative population trend estimates, is more appropriate for nomads than static geographic range size estimation based on pooled occurrences across time, and can be used for classification of extinction risk for nomadic species anywhere that sufficient occurrence data have been collected to derive species distribution models.

Extinction risk in a nomadic species as measured by minimum AOO is not necessarily the same as that of an otherwise identical sedentary species. Although a nomad and an equivalent sedentary species could be at equally high risk from threats whilst occupying a bottleneck or refugial site, the ability of nomads to expand in distribution (and population) when environmental conditions improve may buffer them from stochastic threats over the long term because they can move on and take advantage of good conditions elsewhere (Dean 2004). However, recent work shows that the buffering effect of movements are obviated in the face of widespread habitat loss; equal declines are observed among migrants and nonmigrants in Australia and the United States (Albright et al. 2010; Bennett et al. 2014). Movement itself could also be risky in the sense that locations and timings of suitable resources are unpredictable and irregular (Mac Nally et al. 2009). Additionally, in some cases, threats can be concentrated in precisely the areas to which nomadic species contract (Stojanovic et al. 2014). For example, both invasive predators and livestock grazing follow rainfall patterns during prolonged drought (Reid & Fleming 1992; Greenville et al. 2014).

Although nomads are often wide-ranging, they are rarely habitat generalists. Nomads instead can be highly habitat specific, keying into specific environmental conditions such as a vegetation seeding or flowering events (e.g., Pavey & Nano 2013; Tischler et al. 2013; Webb et al. 2014), which makes them less resilient to environmental change than sedentary generalist species. There has been widespread modification and transformation of vegetation across inland Australia; 46% of the continent is subject to grazing of native vegetation (SoE 2011), and this is likely to have affected nomadic birds (Reid & Fleming 1992).

**Figure 5:**
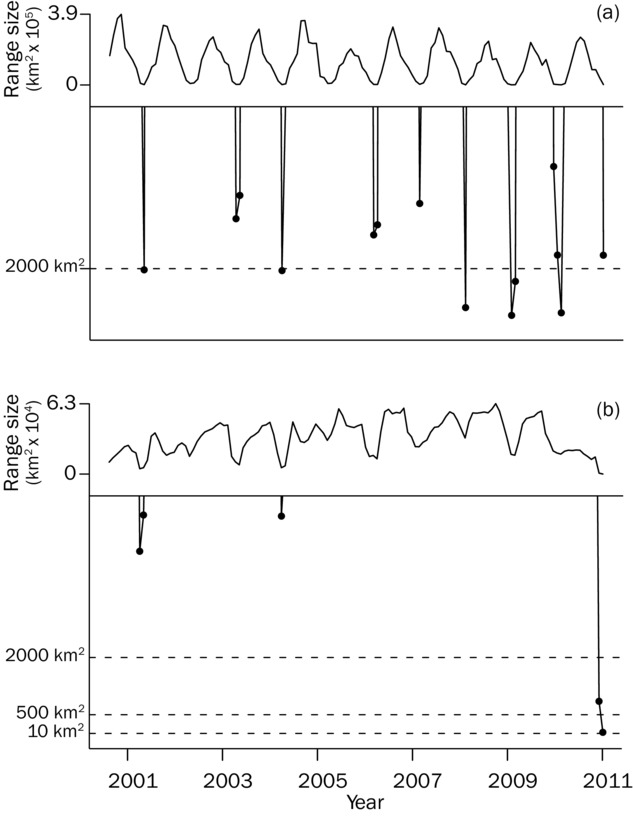
Geographic range size dynamics for (a) Scarlet-chested Parrot and (b) Chestnut-breasted Whiteface. Dashed lines indicate thresholds under IUCN Red List guidelines B2ii (area of occupancy: critically endangered, <10 km^2^; endangered, <500 km^2^; vulnerable, < 2000 km^2^), and the minima are magnified below each plot.

Our data suggest that threat assessments (e.g., IUCN red listing) based on geographic range size may underestimate extinction risk in nomadic species if such assessments are based on pooled occurrences across time. Populations of nomadic species might rarely cover the pooled geographic range, instead frequently contracting to areas significantly smaller than their maximal distribution. For instance, the Scarlet-chested Parrot is currently listed as least concern because the population is thought to be stable and occupy a large area (EOO 262,000 km^2^; BirdLife International 2013), though the accuracy of population estimates is acknowledged to be poor. However, given the evidence of extreme fluctuations in geographic range size presented here (Fig.2) and the repeated occurrence of minimum AOO below the 2000 km^2^ IUCN vulnerable threshold (Fig.5a; IUCN 2014), there is perhaps a case to increase the threat category of this species. Similarly, our models hint at strong fluctuations in geographic distribution for the Chestnut-breasted Whiteface (Fig.2) and that the AOO for this species may drop to 37 km^2^ at certain times, which is well below the IUCN endangered threshold (Fig.5b) (IUCN Red List criteria B2: AOO < 500 km^2^). These examples suggest that species may be at greater risk of extinction than suggested by their current IUCN status, and we urge field researchers to look for empirical evidence of distributional fluctuations.

Which measure of geographic range size best reflects an appropriate measure of extinction risk for nomadic species? Fluctuation in population size is already captured under criterion B2cii (IUCN Red List), but it only applies if absolute area thresholds in EOO or AOO have been breached (IUCN 2014), and there are no guidelines around fluctuating range size. Guidelines indicate that for migratory species, the geographic range size metric for red listing should be based on the smaller of either the breeding or nonbreeding distributions (IUCN 2014). Although recognizing it is not a direct analogy, we suggest assessing extinction risk for nomads on the basis of minimum range size, either observed or estimated, in situations where a species cannot be assessed using alternative methods such as fluctuations in population size. Our approach assumes that the summed environmental suitability in occupied areas represents a species’ geographic range size, which although parsimonious in the absence of data to the contrary, would benefit from detailed investigation. Although the true relationship between fluctuating distributions and extinction risk is unresolved for nomads, we assume that the relationships among environmental suitability, population density, and extinction risk are linear.

Nomadic movements across space and time limit our ability to determine population dynamics and consequently our ability to estimate risk on that basis. Many migratory species can be surveyed annually because of predictable movements to and from breeding grounds, which allows reasonably accurate measurement of population change and extinction risk (Wilson et al. 2011; Clemens et al. 2012). However, for nomadic species when and where we monitor may dramatically influence our estimates of both population abundance and trend. Figure3 illustrates a possible outcome of monitoring at different locations across a nomadic species’ distribution, assuming for the purpose of this example a linear relationship between environmental suitability and population size (Lawton 1993). Extrapolating trends measured at the center of a distribution could lead to an overestimate of total population size and an underestimate of population fluctuations. Conversely, monitoring at the edge of the range could indicate a dramatically fluctuating population, with low to medium probability of presence, depending on the location monitored. The overall trend (Fig.3a) shows population size and dynamics may be somewhere between those estimated by monitoring at the core (Fig.3b) and edges (Fig.3c-d), consistent with the general pattern that populations are more abundant at the center of their ranges and variable toward range edges (Brown 1984; Gaston 2003). It would be very difficult to identify any underlying population trend in the presence of such complex spatial and temporal fluctuations. Geographic range size determination thus seems the most tractable way to assess extinction risk in nomadic species, despite its reliance on a (as yet untested) theoretical relationship between environmental suitability and population size.

Although our models enhance the capability to estimate extinction metrics, it is unclear how distribution fluctuations impact long-term persistence. The impact of fluctuations on population persistence is a function of the number of subpopulations and the synchronicity of fluctuation across those populations (Lawton et al. 1994). Both theory and empirical evidence predict that extinction risk is higher in species with highly fluctuating populations (Pimm et al. 1988; Hung et al. 2014), yet such fluctuations could also indicate an ability to cope with changing patterns of resources in a landscape. Although many nomadic species are hypothesized to have an inherent capacity to bounce back from spatial and numerical bottlenecks (Dean 2004; Jonzén et al. 2011), we know little about their vulnerability to environmental change. The response to bottlenecks may be related to the length and amplitude of the bottleneck and the presence and condition of refugia (Mangel & Tier 1994). For instance, an extreme drought in eastern Australia in 1902 led to mass mortality among birds in central Queensland that persisted for many years and was a major contributor to the extinction of the once common Paradise Parakeet (*Psephotus pulcherrimus*) (Keast 1959), whose refugial grounds had been lost to newly expanding agriculture. Similarly, short-term heat waves can cause huge mortalities in arid-zone birds. One such event occurred in January 2009, when temperatures rose above 45 °C for several consecutive days and killed thousands of birds (McKechnie et al. 2012). Predicted increases in heat wave frequency may exacerbate the impact of such mortality events (McKechnie & Wolf 2010). Cooler microclimates can mediate these mortalities, and conservation actions for susceptible species may include provision of shaded bird-accessible water points (McKechnie et al. 2012). These species evolved in a landscape where environmental conditions are dynamic, and strategies such as opportunistic breeding and diet switching may facilitate the ability of arid-zone birds to recover from bottlenecks (Dean 2004). However, rapid environmental change such as climate change has the potential to outpace species’ abilities to respond to temporally and spatially variable environmental conditions. Further research is required to determine the thresholds beyond which the ability of these species to recover from temporal, spatial, and evolutionary bottlenecks is impaired.

By generating estimates of both mean and minimum range size across time, our study shows how to derive more accurate empirical estimates of fluctuations in dynamic species than those currently available. Truly accurate estimation of long-term persistence in nomads such as arid-zone birds is limited by our lack of knowledge of the impact of human land use change and the ability of species to overcome environmental fluctuations. In the absence of such information, our approach provides a valuable starting point for conservation planning for dynamic species.

## Supporting Information

Disclaimer: Supplementary materials have been peer-reviewed but not copyedited.

Information on vegetation reclassification (Appendix S1) and range size metrics and model statistics (Appendix S2); plots of temporal dynamics in range size (Appendix S3); and animated maps of environmental suitability over time (Appendix S4) are available online for all 43 modeled species. The authors are solely responsible for the content and functionality of these materials. Queries (other than absence of the material) should be directed to the corresponding author.
