# Supplementary material for: Geographic range size and extinction risk assessment in nomadic species
Source: Conserv Biol. 2015 Jan 9;29(3):865–76. doi: 10.1111/cobi.12440 (PMC4681363; doi:10.1111/cobi.12440)
Supplement: Supplementary file 1 — Information on vegetation reclassification (Appendix S1) and range size metrics and model statistics (Appendix S2); plots of temporal dynamics in range size (Appendix S3); and animated maps of environmental suitability over time (Appendix S4) are available online for all 43 modeled species. The authors are solely responsible for the content and functionality of these materials. Queries (other than absence of the material) should be directed to the corresponding author. [file cobi0029-0865-sd1.zip › cobi12440-sup-0003-text-Table_S2.docx]

## Table S2

*Table S2: Species movement classifications, modelled range size metrics and model validation statistics for 43 Australian nomadic birds.*

| Species | Movement status | Overall range size (km2) | Minimum range size (km^2^) | Mean range size (km^2^) | Maximum range size (km^2^) | Standard deviation of the mean range size | Coefficient of variation | Magnitude of fluctuation in range size | Difference between model and null AUC | Number of occurrences used in models |
| --- | --- | --- | --- | --- | --- | --- | --- | --- | --- | --- |
| Stubble Quail *Coturnix pectoralis* | Nomadic | 1,819,376 | 169,017 | 435,663 | 1,199,518 | 171,941 | 0.395 | 7 | 0.140 | 472 |
| Black-shouldered Kite *Elanus axillaris* | Nomadic | 2,645,411 | 113,305 | 462,933 | 1,650,596 | 360,543 | 0.779 | 15 | 0.181 | 2845 |
| Letter-winged Kite *Elanus scriptus* | Nomadic | 719,691 | 60,454 | 202,000 | 618,280 | 116,531 | 0.577 | 10 | 0.110 | 29 |
| Spotted Harrier *Circus assimilis* | Nomadic | 3,559,606 | 583,026 | 1,316,650 | 2,554,575 | 391,594 | 0.297 | 4 | 0.182 | 1255 |
| Australian Bustard *Ardeotis australis* | Nomadic | 3,135,949 | 1,123,919 | 1,626,294 | 2,349,761 | 293,412 | 0.180 | 2 | 0.214 | 1311 |
| Common Bronzewing *Phaps chalcoptera* | Possibly nomadic | 1,097,672 | 86,879 | 310,034 | 531,379 | 108,800 | 0.351 | 6 | 0.136 | 5347 |
| Flock Bronzewing *Phaps histrionica* | Nomadic | 916,107 | 84,554 | 302,802 | 689,112 | 134,677 | 0.445 | 8 | 0.194 | 165 |
| Diamond Dove *Geopelia cuneata* | Nomadic | 2,731,995 | 220,878 | 1,134,687 | 2,028,898 | 416,395 | 0.367 | 9 | 0.236 | 4380 |
| Grey Falcon *Falco hypoleucos* | Nomadic | 2,572,585 | 882,558 | 1,229,142 | 1,515,573 | 144,041 | 0.117 | 2 | 0.109 | 138 |
| Black Falcon *Falco subniger* | Nomadic | 2,675,534 | 537,230 | 1,011,626 | 1,698,811 | 234,623 | 0.232 | 3 | 0.093 | 472 |
| Major Mitchell's Cockatoo *Lophochroa leadbeateri* | Possibly nomadic | 2,404,222 | 560,730 | 942,133 | 1,519,569 | 189,782 | 0.201 | 3 | 0.244 | 1137 |
| Cockatiel *Nymphicus hollandicus* | Nomadic | 3,270,352 | 106,111 | 987,539 | 1,858,621 | 406,414 | 0.412 | 18 | 0.164 | 4597 |
| Bourke's Parrot *Neopsephotus bourkii* | Nomadic | 1,657,523 | 746,496 | 975,446 | 1,233,106 | 103,664 | 0.106 | 2 | 0.214 | 308 |
| Scarlet-chested Parrot *Neophema splendida* | Nomadic | 496,793 | 776 | 128,339 | 389,329 | 100,571 | 0.784 | 502 | 0.195 | 69 |
| Budgerigar *Melopsittacus undulatus* | Nomadic | 2,789,945 | 186,998 | 1,133,398 | 2,083,904 | 388,315 | 0.343 | 11 | 0.228 | 5492 |
| Black Honeyeater *Sugomel nigrum* | Nomadic | 2,206,769 | 237,940 | 890,360 | 1,628,696 | 263,342 | 0.296 | 7 | 0.264 | 917 |
| Pied Honeyeater *Certhionyx variegatus* | Nomadic | 2,538,637 | 630,913 | 1,140,775 | 1,918,270 | 365,582 | 0.320 | 3 | 0.272 | 1202 |
| Brown Honeyeater *Lichmera indistincta* | Nomadic | 2,571,125 | 138,958 | 871,046 | 1,602,178 | 385,477 | 0.443 | 12 | 0.189 | 8553 |
| Painted Honeyeater *Grantiella picta* | Nomadic | 780,039 | 92,922 | 234,556 | 399,436 | 89,267 | 0.381 | 4 | 0.152 | 190 |
| Striped Honeyeater *Plectorhyncha lanceolata* | Possibly nomadic | 659,307 | 82,817 | 252,542 | 414,736 | 81,228 | 0.322 | 5 | 0.239 | 3092 |
| Gibberbird *Ashbyia lovensis* | Nomadic | 327,149 | 151,157 | 189,748 | 223,760 | 14,209 | 0.075 | 1 | 0.181 | 138 |
| Crimson Chat *Epthianura tricolor* | Nomadic | 2,611,986 | 157,107 | 1,151,709 | 2,039,803 | 468,049 | 0.406 | 13 | 0.260 | 2517 |
| Orange Chat *Epthianura aurifrons* | Nomadic | 2,138,565 | 493,032 | 1,059,164 | 1,546,689 | 225,849 | 0.213 | 3 | 0.264 | 873 |
| Yellow Chat *Epthianura crocea* | Nomadic | 257,089 | 26,570 | 72,656 | 143,560 | 23,518 | 0.324 | 5 | 0.188 | 142 |
| White-fronted Chat *Epthianura albifrons* | Nomadic | 625,249 | 64,954 | 207,426 | 383,826 | 63,656 | 0.307 | 6 | 0.255 | 1408 |
| Grey Honeyeater *Conopophila whitei* | Nomadic | 1,297,181 | 108,314 | 573,783 | 1,076,109 | 190,734 | 0.332 | 10 | 0.160 | 47 |
| Spiny-cheeked Honeyeater *Acanthagenys rufogularis* | Possibly nomadic | 2,063,826 | 448,022 | 932,766 | 1,573,907 | 224,940 | 0.241 | 4 | 0.120 | 14872 |
| White-fronted Honeyeater *Purnella albifrons* | Nomadic | 1,669,300 | 103,538 | 375,174 | 1,087,753 | 235,236 | 0.627 | 11 | 0.278 | 4090 |
| Grey-headed Honeyeater *Ptilotula keartlandi* | Possibly nomadic | 1,814,667 | 185,357 | 632,193 | 1,153,436 | 186,643 | 0.295 | 6 | 0.325 | 1960 |
| Grey-fronted Honeyeater *Ptilotula plumula* | Nomadic | 2,210,412 | 255,598 | 708,074 | 1,192,172 | 250,579 | 0.354 | 5 | 0.287 | 1617 |
| Striated Pardalote *Pardalotus striatus* | Possibly nomadic | 1,161,005 | 219,578 | 408,166 | 587,510 | 71,184 | 0.174 | 3 | 0.075 | 20315 |
| Western Gerygone *Gerygone fusca* | Nomadic | 2,271,607 | 384,749 | 754,571 | 1,514,307 | 220,732 | 0.293 | 4 | 0.201 | 1747 |
| Chestnut-breasted Whiteface *Aphelocephala pectoralis* | Possibly nomadic | 71,193 | 37 | 33,352 | 63,160 | 15,247 | 0.457 | 1720 | 0.227 | 68 |
| Banded Whiteface *Aphelocephala nigricincta* | Possibly nomadic | 1,446,464 | 336,688 | 644,626 | 883,714 | 117,342 | 0.182 | 3 | 0.249 | 330 |
| Ground Cuckooshrike *Coracina maxima* | Nomadic | 3,155,208 | 448,945 | 1,379,621 | 2,109,783 | 375,612 | 0.272 | 5 | 0.163 | 572 |
| Grey Fantail *Rhipidura albiscapa* | Nomadic | 436,107 | 86,055 | 166,907 | 299,711 | 42,732 | 0.256 | 3 | 0.164 | 8239 |
| Little Crow *Corvus bennetti* | Nomadic | 2,508,774 | 1,111,711 | 1,636,675 | 2,047,464 | 195,665 | 0.120 | 2 | 0.225 | 3395 |
| Jacky Winter *Microeca fascinans* | Possibly nomadic | 1,564,910 | 326,901 | 580,638 | 933,210 | 127,589 | 0.220 | 3 | 0.167 | 6276 |
| Red-capped Robin *Petroica goodenovii* | Possibly nomadic | 2,829,147 | 562,818 | 1,288,807 | 2,198,158 | 334,505 | 0.260 | 4 | 0.135 | 7089 |
| Mistletoebird *Dicaeum hirundinaceum* | Possibly nomadic | 2,873,533 | 336,324 | 922,454 | 1,824,905 | 339,363 | 0.368 | 5 | 0.101 | 9021 |
| Painted Finch *Emblema pictum* | Possibly nomadic | 1,494,337 | 350,768 | 686,168 | 1,144,220 | 174,715 | 0.255 | 3 | 0.265 | 521 |
| Plum-headed Finch *Neochmia modesta* | Nomadic | 955,399 | 89,282 | 328,518 | 621,434 | 97,423 | 0.297 | 7 | 0.184 | 257 |
| Pictorella Mannikin *Heteromunia pectoralis* | Nomadic | 1,284,739 | 33,227 | 302,550 | 870,705 | 205,929 | 0.681 | 26 | 0.235 | 249 |
