# Supplementary figures and images for: Geographic range size and extinction risk assessment in nomadic species
Source: Conserv Biol. 2015 Jan 9;29(3):865–76. doi: 10.1111/cobi.12440 (PMC4681363; doi:10.1111/cobi.12440)

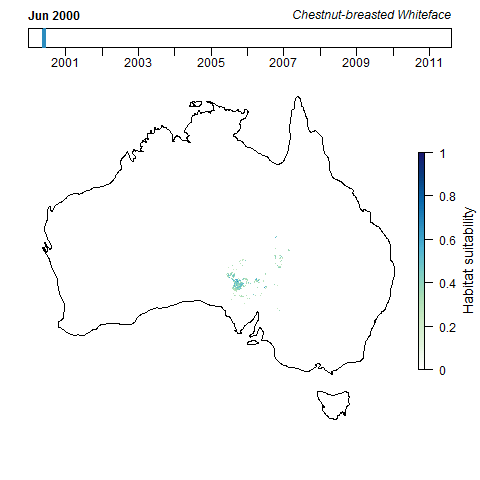

Supplement: Supplementary file 1 — Information on vegetation reclassification (Appendix S1) and range size metrics and model statistics (Appendix S2); plots of temporal dynamics in range size (Appendix S3); and animated maps of environmental suitability over time (Appendix S4) are available online for all 43 modeled species. The authors are solely responsible for the content and functionality of these materials. Queries (other than absence of the material) should be directed to the corresponding author. [file cobi0029-0865-sd1.zip › Supplementary-S4-Animations/Aphelocephala_pectoralis.gif]

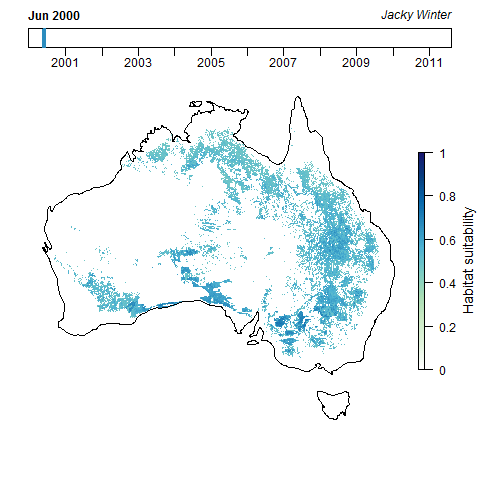

Supplement: Supplementary file 1 — Information on vegetation reclassification (Appendix S1) and range size metrics and model statistics (Appendix S2); plots of temporal dynamics in range size (Appendix S3); and animated maps of environmental suitability over time (Appendix S4) are available online for all 43 modeled species. The authors are solely responsible for the content and functionality of these materials. Queries (other than absence of the material) should be directed to the corresponding author. [file cobi0029-0865-sd1.zip › Supplementary-S4-Animations/Microeca_fascinans.gif]

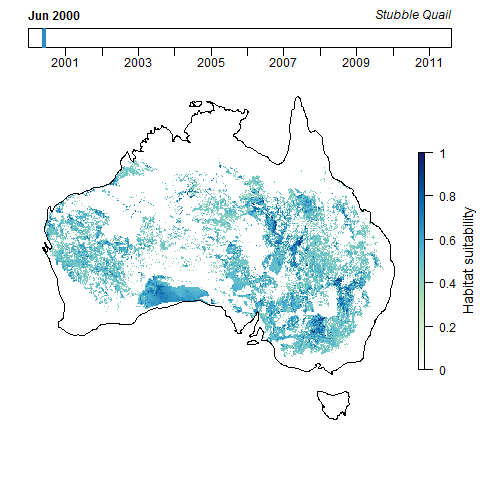

Supplement: Supplementary file 1 — Information on vegetation reclassification (Appendix S1) and range size metrics and model statistics (Appendix S2); plots of temporal dynamics in range size (Appendix S3); and animated maps of environmental suitability over time (Appendix S4) are available online for all 43 modeled species. The authors are solely responsible for the content and functionality of these materials. Queries (other than absence of the material) should be directed to the corresponding author. [file cobi0029-0865-sd1.zip › Supplementary-S4-Animations/Coturnix_pectoralis.gif]

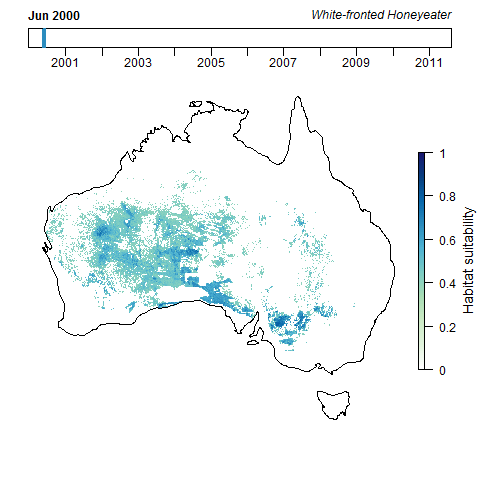

Supplement: Supplementary file 1 — Information on vegetation reclassification (Appendix S1) and range size metrics and model statistics (Appendix S2); plots of temporal dynamics in range size (Appendix S3); and animated maps of environmental suitability over time (Appendix S4) are available online for all 43 modeled species. The authors are solely responsible for the content and functionality of these materials. Queries (other than absence of the material) should be directed to the corresponding author. [file cobi0029-0865-sd1.zip › Supplementary-S4-Animations/Purnella_albifrons.gif]

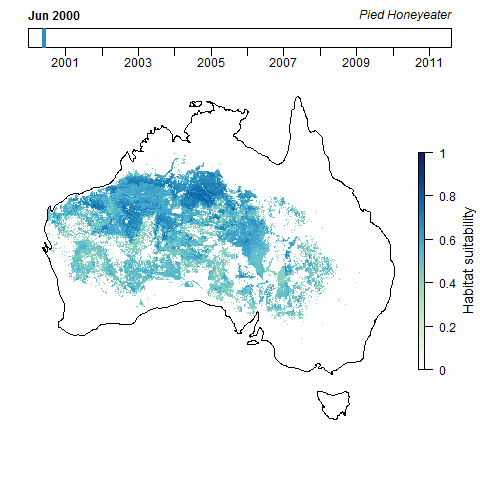

Supplement: Supplementary file 1 — Information on vegetation reclassification (Appendix S1) and range size metrics and model statistics (Appendix S2); plots of temporal dynamics in range size (Appendix S3); and animated maps of environmental suitability over time (Appendix S4) are available online for all 43 modeled species. The authors are solely responsible for the content and functionality of these materials. Queries (other than absence of the material) should be directed to the corresponding author. [file cobi0029-0865-sd1.zip › Supplementary-S4-Animations/Certhionyx_variegatus.gif]

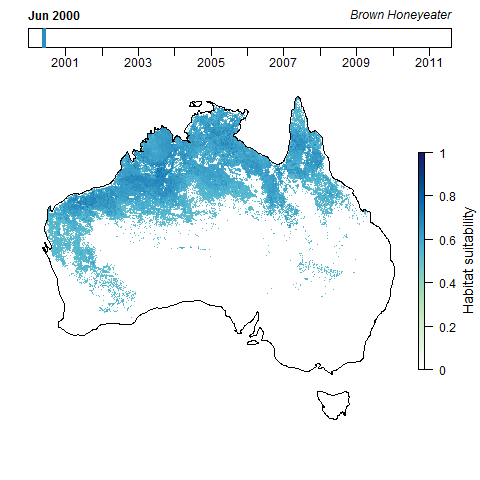

Supplement: Supplementary file 1 — Information on vegetation reclassification (Appendix S1) and range size metrics and model statistics (Appendix S2); plots of temporal dynamics in range size (Appendix S3); and animated maps of environmental suitability over time (Appendix S4) are available online for all 43 modeled species. The authors are solely responsible for the content and functionality of these materials. Queries (other than absence of the material) should be directed to the corresponding author. [file cobi0029-0865-sd1.zip › Supplementary-S4-Animations/Lichmera_indistincta.gif]

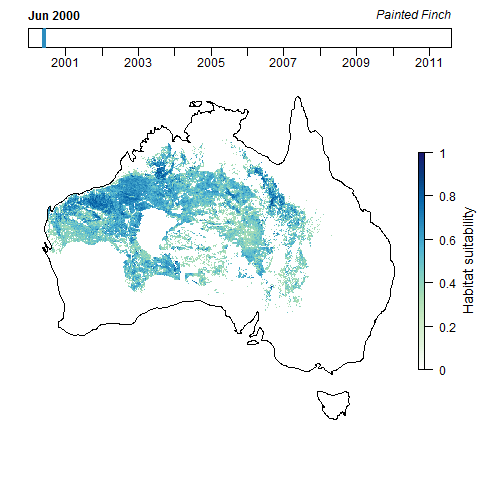

Supplement: Supplementary file 1 — Information on vegetation reclassification (Appendix S1) and range size metrics and model statistics (Appendix S2); plots of temporal dynamics in range size (Appendix S3); and animated maps of environmental suitability over time (Appendix S4) are available online for all 43 modeled species. The authors are solely responsible for the content and functionality of these materials. Queries (other than absence of the material) should be directed to the corresponding author. [file cobi0029-0865-sd1.zip › Supplementary-S4-Animations/Emblema_pictum.gif]

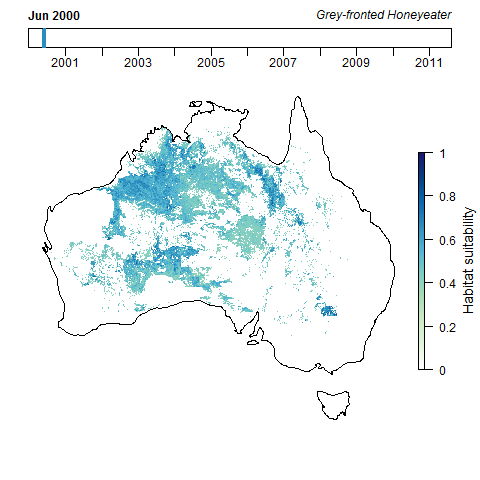

Supplement: Supplementary file 1 — Information on vegetation reclassification (Appendix S1) and range size metrics and model statistics (Appendix S2); plots of temporal dynamics in range size (Appendix S3); and animated maps of environmental suitability over time (Appendix S4) are available online for all 43 modeled species. The authors are solely responsible for the content and functionality of these materials. Queries (other than absence of the material) should be directed to the corresponding author. [file cobi0029-0865-sd1.zip › Supplementary-S4-Animations/Lichenostomus_plumulus.gif]

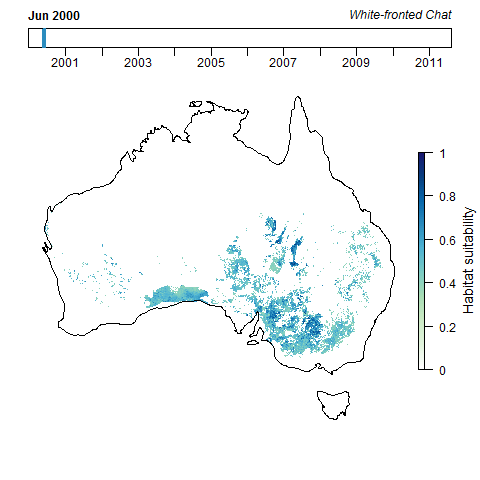

Supplement: Supplementary file 1 — Information on vegetation reclassification (Appendix S1) and range size metrics and model statistics (Appendix S2); plots of temporal dynamics in range size (Appendix S3); and animated maps of environmental suitability over time (Appendix S4) are available online for all 43 modeled species. The authors are solely responsible for the content and functionality of these materials. Queries (other than absence of the material) should be directed to the corresponding author. [file cobi0029-0865-sd1.zip › Supplementary-S4-Animations/Epthianura_albifrons.gif]

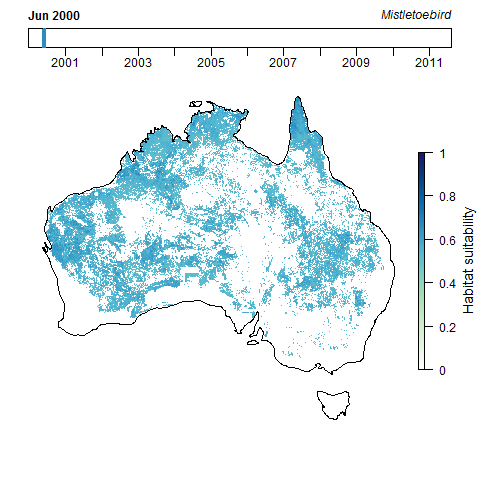

Supplement: Supplementary file 1 — Information on vegetation reclassification (Appendix S1) and range size metrics and model statistics (Appendix S2); plots of temporal dynamics in range size (Appendix S3); and animated maps of environmental suitability over time (Appendix S4) are available online for all 43 modeled species. The authors are solely responsible for the content and functionality of these materials. Queries (other than absence of the material) should be directed to the corresponding author. [file cobi0029-0865-sd1.zip › Supplementary-S4-Animations/Dicaeum_hirundinaceum.gif]

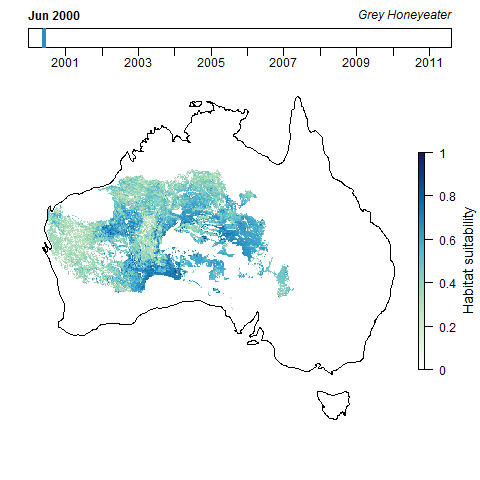

Supplement: Supplementary file 1 — Information on vegetation reclassification (Appendix S1) and range size metrics and model statistics (Appendix S2); plots of temporal dynamics in range size (Appendix S3); and animated maps of environmental suitability over time (Appendix S4) are available online for all 43 modeled species. The authors are solely responsible for the content and functionality of these materials. Queries (other than absence of the material) should be directed to the corresponding author. [file cobi0029-0865-sd1.zip › Supplementary-S4-Animations/Conopophila_whitei.gif]

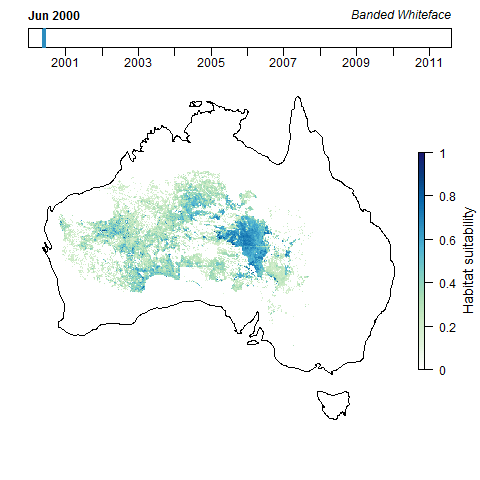

Supplement: Supplementary file 1 — Information on vegetation reclassification (Appendix S1) and range size metrics and model statistics (Appendix S2); plots of temporal dynamics in range size (Appendix S3); and animated maps of environmental suitability over time (Appendix S4) are available online for all 43 modeled species. The authors are solely responsible for the content and functionality of these materials. Queries (other than absence of the material) should be directed to the corresponding author. [file cobi0029-0865-sd1.zip › Supplementary-S4-Animations/Aphelocephala_nigricincta.gif]

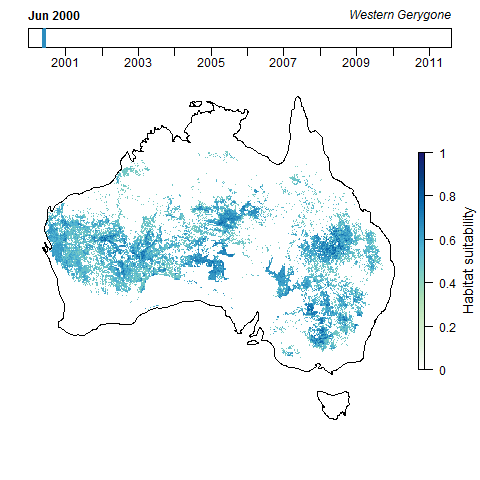

Supplement: Supplementary file 1 — Information on vegetation reclassification (Appendix S1) and range size metrics and model statistics (Appendix S2); plots of temporal dynamics in range size (Appendix S3); and animated maps of environmental suitability over time (Appendix S4) are available online for all 43 modeled species. The authors are solely responsible for the content and functionality of these materials. Queries (other than absence of the material) should be directed to the corresponding author. [file cobi0029-0865-sd1.zip › Supplementary-S4-Animations/Gerygone_fusca.gif]

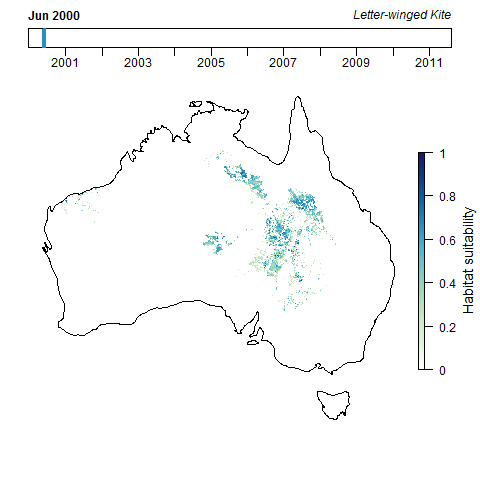

Supplement: Supplementary file 1 — Information on vegetation reclassification (Appendix S1) and range size metrics and model statistics (Appendix S2); plots of temporal dynamics in range size (Appendix S3); and animated maps of environmental suitability over time (Appendix S4) are available online for all 43 modeled species. The authors are solely responsible for the content and functionality of these materials. Queries (other than absence of the material) should be directed to the corresponding author. [file cobi0029-0865-sd1.zip › Supplementary-S4-Animations/Elanus_scriptus.gif]

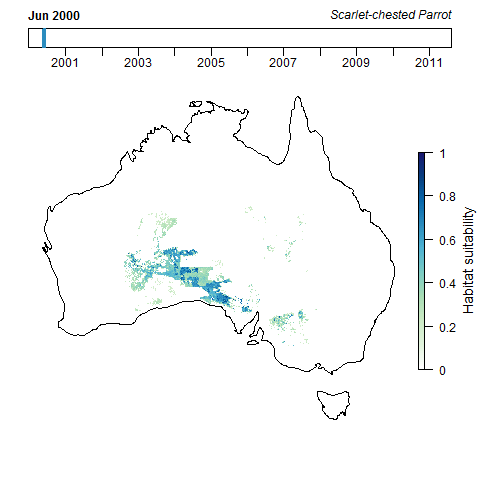

Supplement: Supplementary file 1 — Information on vegetation reclassification (Appendix S1) and range size metrics and model statistics (Appendix S2); plots of temporal dynamics in range size (Appendix S3); and animated maps of environmental suitability over time (Appendix S4) are available online for all 43 modeled species. The authors are solely responsible for the content and functionality of these materials. Queries (other than absence of the material) should be directed to the corresponding author. [file cobi0029-0865-sd1.zip › Supplementary-S4-Animations/Neophema_splendida.gif]

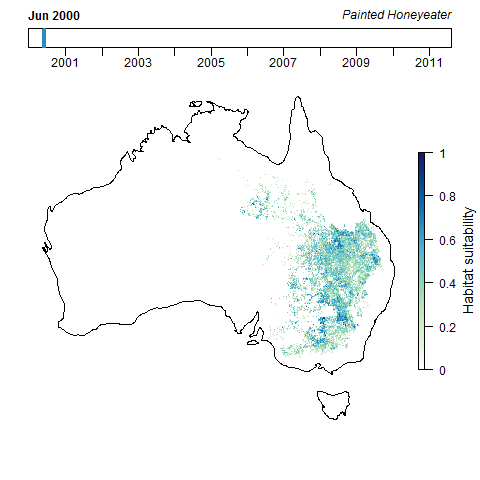

Supplement: Supplementary file 1 — Information on vegetation reclassification (Appendix S1) and range size metrics and model statistics (Appendix S2); plots of temporal dynamics in range size (Appendix S3); and animated maps of environmental suitability over time (Appendix S4) are available online for all 43 modeled species. The authors are solely responsible for the content and functionality of these materials. Queries (other than absence of the material) should be directed to the corresponding author. [file cobi0029-0865-sd1.zip › Supplementary-S4-Animations/Grantiella_picta.gif]

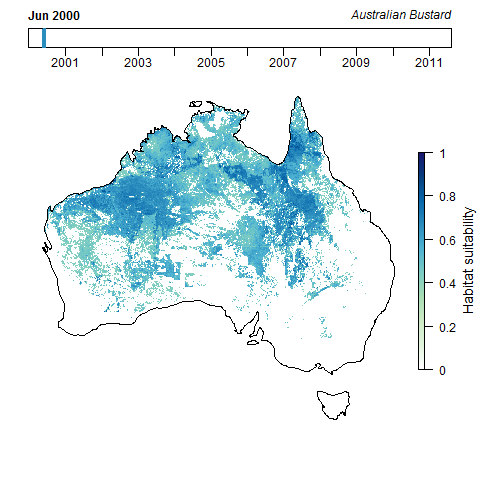

Supplement: Supplementary file 1 — Information on vegetation reclassification (Appendix S1) and range size metrics and model statistics (Appendix S2); plots of temporal dynamics in range size (Appendix S3); and animated maps of environmental suitability over time (Appendix S4) are available online for all 43 modeled species. The authors are solely responsible for the content and functionality of these materials. Queries (other than absence of the material) should be directed to the corresponding author. [file cobi0029-0865-sd1.zip › Supplementary-S4-Animations/Ardeotis_australis.gif]

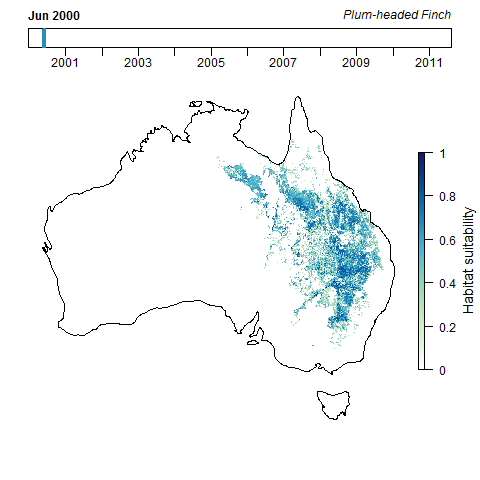

Supplement: Supplementary file 1 — Information on vegetation reclassification (Appendix S1) and range size metrics and model statistics (Appendix S2); plots of temporal dynamics in range size (Appendix S3); and animated maps of environmental suitability over time (Appendix S4) are available online for all 43 modeled species. The authors are solely responsible for the content and functionality of these materials. Queries (other than absence of the material) should be directed to the corresponding author. [file cobi0029-0865-sd1.zip › Supplementary-S4-Animations/Neochmia_modesta.gif]

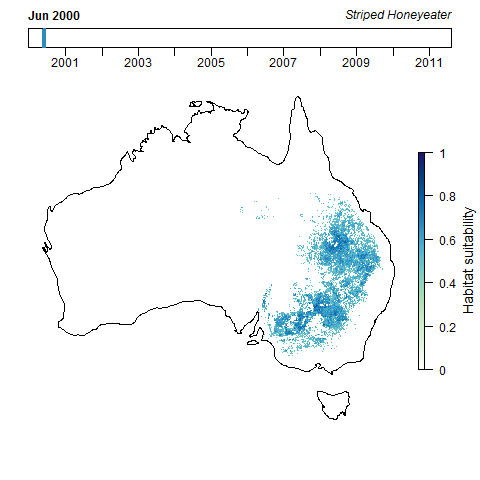

Supplement: Supplementary file 1 — Information on vegetation reclassification (Appendix S1) and range size metrics and model statistics (Appendix S2); plots of temporal dynamics in range size (Appendix S3); and animated maps of environmental suitability over time (Appendix S4) are available online for all 43 modeled species. The authors are solely responsible for the content and functionality of these materials. Queries (other than absence of the material) should be directed to the corresponding author. [file cobi0029-0865-sd1.zip › Supplementary-S4-Animations/Plectorhyncha_lanceolata.gif]

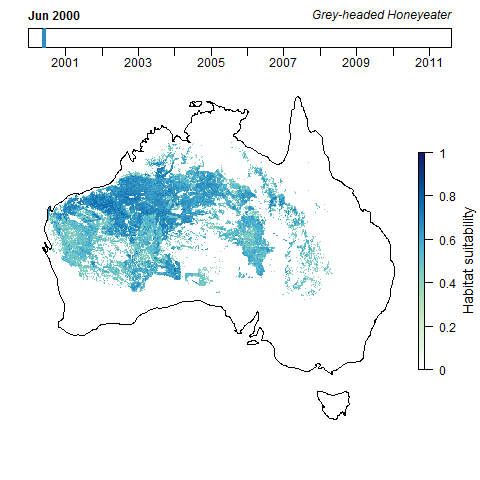

Supplement: Supplementary file 1 — Information on vegetation reclassification (Appendix S1) and range size metrics and model statistics (Appendix S2); plots of temporal dynamics in range size (Appendix S3); and animated maps of environmental suitability over time (Appendix S4) are available online for all 43 modeled species. The authors are solely responsible for the content and functionality of these materials. Queries (other than absence of the material) should be directed to the corresponding author. [file cobi0029-0865-sd1.zip › Supplementary-S4-Animations/Lichenostomus_keartlandi.gif]

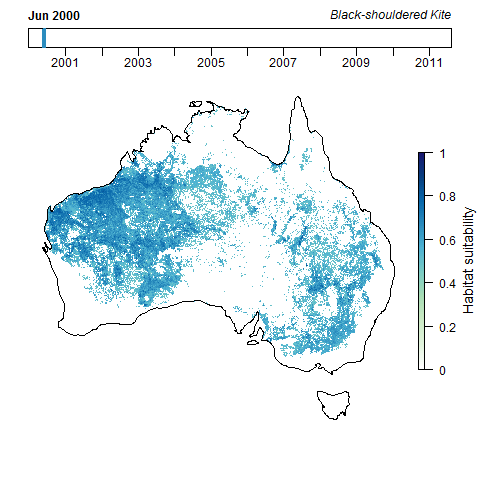

Supplement: Supplementary file 1 — Information on vegetation reclassification (Appendix S1) and range size metrics and model statistics (Appendix S2); plots of temporal dynamics in range size (Appendix S3); and animated maps of environmental suitability over time (Appendix S4) are available online for all 43 modeled species. The authors are solely responsible for the content and functionality of these materials. Queries (other than absence of the material) should be directed to the corresponding author. [file cobi0029-0865-sd1.zip › Supplementary-S4-Animations/Elanus_axillaris.gif]

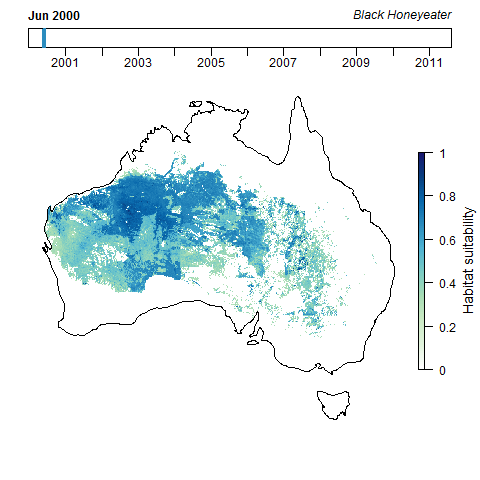

Supplement: Supplementary file 1 — Information on vegetation reclassification (Appendix S1) and range size metrics and model statistics (Appendix S2); plots of temporal dynamics in range size (Appendix S3); and animated maps of environmental suitability over time (Appendix S4) are available online for all 43 modeled species. The authors are solely responsible for the content and functionality of these materials. Queries (other than absence of the material) should be directed to the corresponding author. [file cobi0029-0865-sd1.zip › Supplementary-S4-Animations/Sugomel_niger.gif]

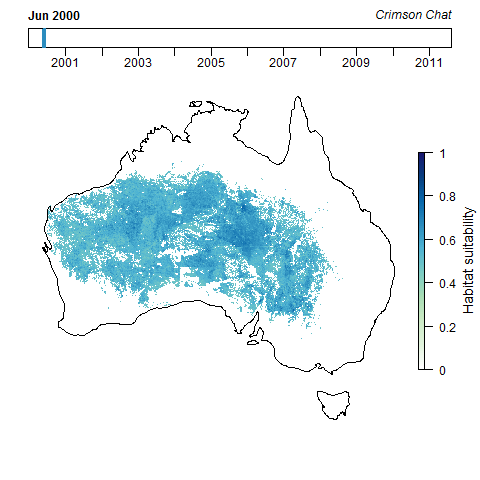

Supplement: Supplementary file 1 — Information on vegetation reclassification (Appendix S1) and range size metrics and model statistics (Appendix S2); plots of temporal dynamics in range size (Appendix S3); and animated maps of environmental suitability over time (Appendix S4) are available online for all 43 modeled species. The authors are solely responsible for the content and functionality of these materials. Queries (other than absence of the material) should be directed to the corresponding author. [file cobi0029-0865-sd1.zip › Supplementary-S4-Animations/Epthianura_tricolor.gif]

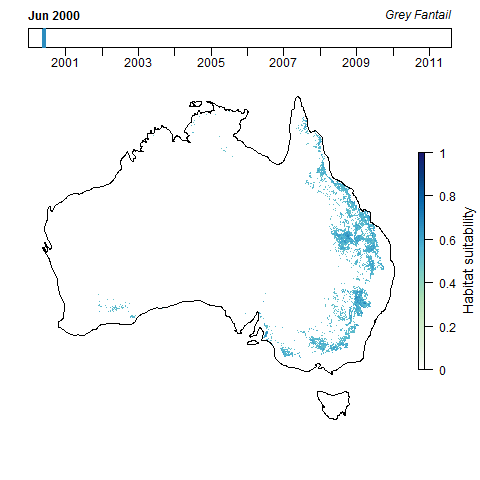

Supplement: Supplementary file 1 — Information on vegetation reclassification (Appendix S1) and range size metrics and model statistics (Appendix S2); plots of temporal dynamics in range size (Appendix S3); and animated maps of environmental suitability over time (Appendix S4) are available online for all 43 modeled species. The authors are solely responsible for the content and functionality of these materials. Queries (other than absence of the material) should be directed to the corresponding author. [file cobi0029-0865-sd1.zip › Supplementary-S4-Animations/Rhipidura_albiscapa.gif]

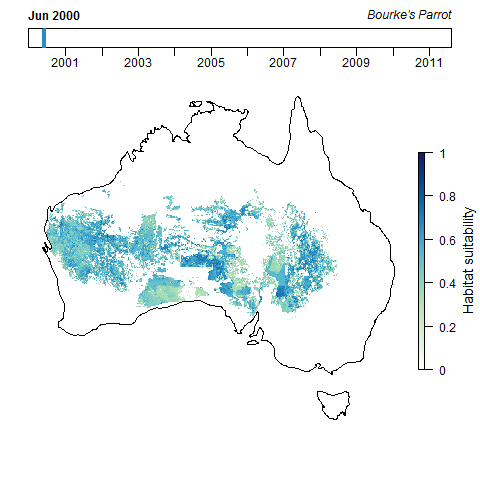

Supplement: Supplementary file 1 — Information on vegetation reclassification (Appendix S1) and range size metrics and model statistics (Appendix S2); plots of temporal dynamics in range size (Appendix S3); and animated maps of environmental suitability over time (Appendix S4) are available online for all 43 modeled species. The authors are solely responsible for the content and functionality of these materials. Queries (other than absence of the material) should be directed to the corresponding author. [file cobi0029-0865-sd1.zip › Supplementary-S4-Animations/Neopsephotus_bourkii.gif]

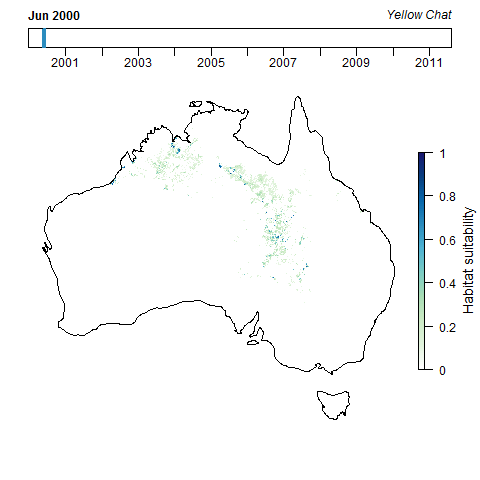

Supplement: Supplementary file 1 — Information on vegetation reclassification (Appendix S1) and range size metrics and model statistics (Appendix S2); plots of temporal dynamics in range size (Appendix S3); and animated maps of environmental suitability over time (Appendix S4) are available online for all 43 modeled species. The authors are solely responsible for the content and functionality of these materials. Queries (other than absence of the material) should be directed to the corresponding author. [file cobi0029-0865-sd1.zip › Supplementary-S4-Animations/Epthianura_crocea.gif]

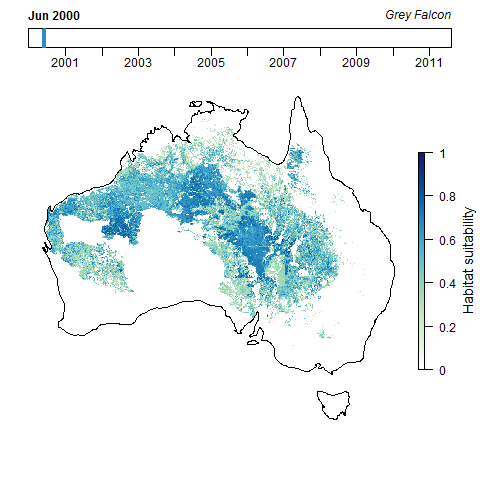

Supplement: Supplementary file 1 — Information on vegetation reclassification (Appendix S1) and range size metrics and model statistics (Appendix S2); plots of temporal dynamics in range size (Appendix S3); and animated maps of environmental suitability over time (Appendix S4) are available online for all 43 modeled species. The authors are solely responsible for the content and functionality of these materials. Queries (other than absence of the material) should be directed to the corresponding author. [file cobi0029-0865-sd1.zip › Supplementary-S4-Animations/Falco_hypoleucos.gif]

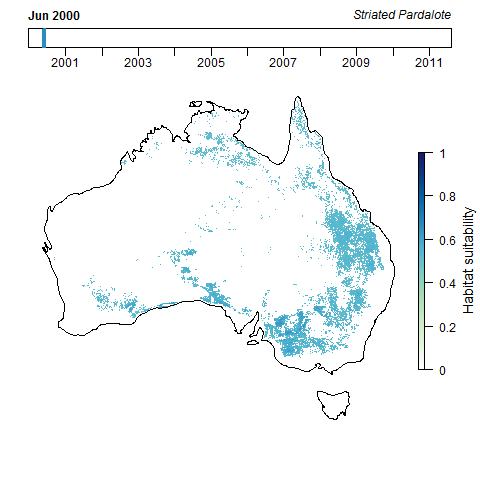

Supplement: Supplementary file 1 — Information on vegetation reclassification (Appendix S1) and range size metrics and model statistics (Appendix S2); plots of temporal dynamics in range size (Appendix S3); and animated maps of environmental suitability over time (Appendix S4) are available online for all 43 modeled species. The authors are solely responsible for the content and functionality of these materials. Queries (other than absence of the material) should be directed to the corresponding author. [file cobi0029-0865-sd1.zip › Supplementary-S4-Animations/Pardalotus_striatus.gif]

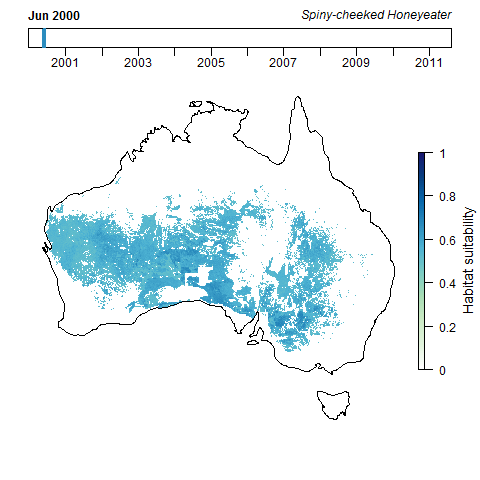

Supplement: Supplementary file 1 — Information on vegetation reclassification (Appendix S1) and range size metrics and model statistics (Appendix S2); plots of temporal dynamics in range size (Appendix S3); and animated maps of environmental suitability over time (Appendix S4) are available online for all 43 modeled species. The authors are solely responsible for the content and functionality of these materials. Queries (other than absence of the material) should be directed to the corresponding author. [file cobi0029-0865-sd1.zip › Supplementary-S4-Animations/Acanthagenys_rufogularis.gif]

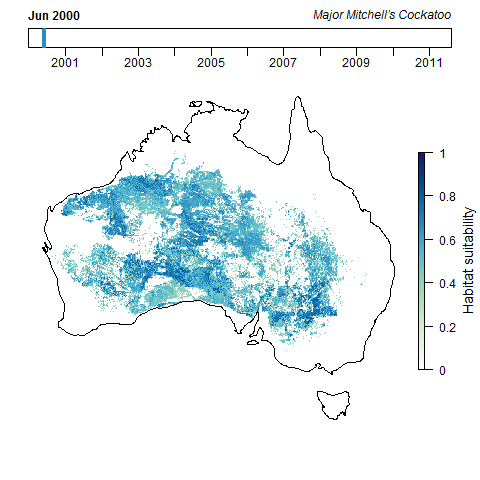

Supplement: Supplementary file 1 — Information on vegetation reclassification (Appendix S1) and range size metrics and model statistics (Appendix S2); plots of temporal dynamics in range size (Appendix S3); and animated maps of environmental suitability over time (Appendix S4) are available online for all 43 modeled species. The authors are solely responsible for the content and functionality of these materials. Queries (other than absence of the material) should be directed to the corresponding author. [file cobi0029-0865-sd1.zip › Supplementary-S4-Animations/Lophochroa_leadbeateri.gif]

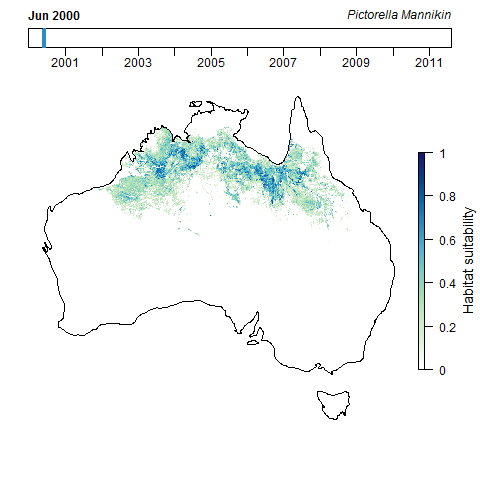

Supplement: Supplementary file 1 — Information on vegetation reclassification (Appendix S1) and range size metrics and model statistics (Appendix S2); plots of temporal dynamics in range size (Appendix S3); and animated maps of environmental suitability over time (Appendix S4) are available online for all 43 modeled species. The authors are solely responsible for the content and functionality of these materials. Queries (other than absence of the material) should be directed to the corresponding author. [file cobi0029-0865-sd1.zip › Supplementary-S4-Animations/Heteromunia_pectoralis.gif]

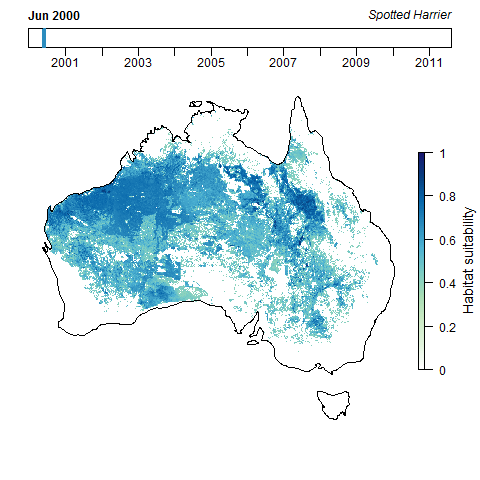

Supplement: Supplementary file 1 — Information on vegetation reclassification (Appendix S1) and range size metrics and model statistics (Appendix S2); plots of temporal dynamics in range size (Appendix S3); and animated maps of environmental suitability over time (Appendix S4) are available online for all 43 modeled species. The authors are solely responsible for the content and functionality of these materials. Queries (other than absence of the material) should be directed to the corresponding author. [file cobi0029-0865-sd1.zip › Supplementary-S4-Animations/Circus_assimilis.gif]

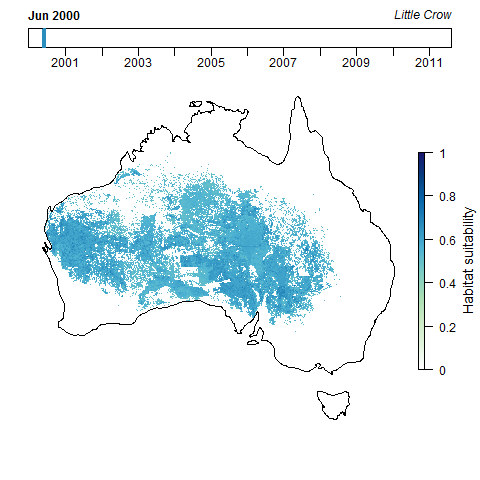

Supplement: Supplementary file 1 — Information on vegetation reclassification (Appendix S1) and range size metrics and model statistics (Appendix S2); plots of temporal dynamics in range size (Appendix S3); and animated maps of environmental suitability over time (Appendix S4) are available online for all 43 modeled species. The authors are solely responsible for the content and functionality of these materials. Queries (other than absence of the material) should be directed to the corresponding author. [file cobi0029-0865-sd1.zip › Supplementary-S4-Animations/Corvus_bennetti.gif]

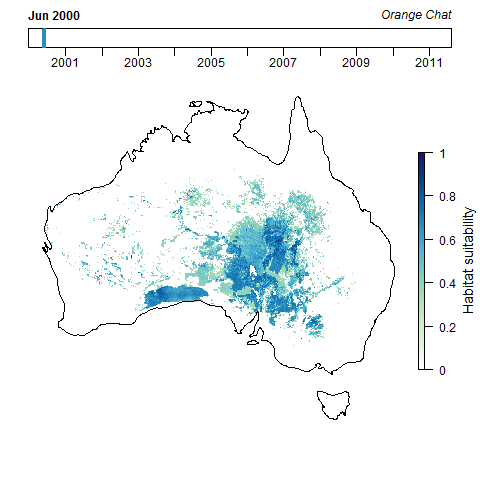

Supplement: Supplementary file 1 — Information on vegetation reclassification (Appendix S1) and range size metrics and model statistics (Appendix S2); plots of temporal dynamics in range size (Appendix S3); and animated maps of environmental suitability over time (Appendix S4) are available online for all 43 modeled species. The authors are solely responsible for the content and functionality of these materials. Queries (other than absence of the material) should be directed to the corresponding author. [file cobi0029-0865-sd1.zip › Supplementary-S4-Animations/Epthianura_aurifrons.gif]

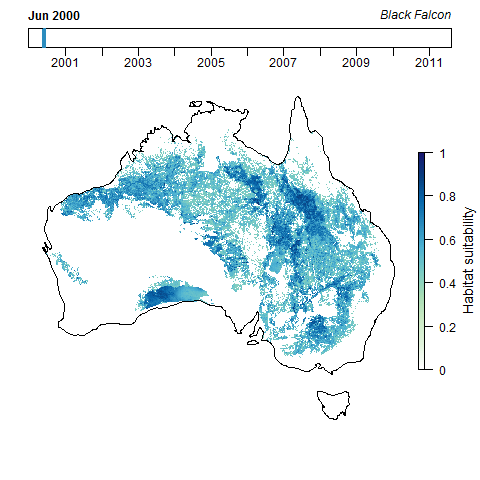

Supplement: Supplementary file 1 — Information on vegetation reclassification (Appendix S1) and range size metrics and model statistics (Appendix S2); plots of temporal dynamics in range size (Appendix S3); and animated maps of environmental suitability over time (Appendix S4) are available online for all 43 modeled species. The authors are solely responsible for the content and functionality of these materials. Queries (other than absence of the material) should be directed to the corresponding author. [file cobi0029-0865-sd1.zip › Supplementary-S4-Animations/Falco_subniger.gif]

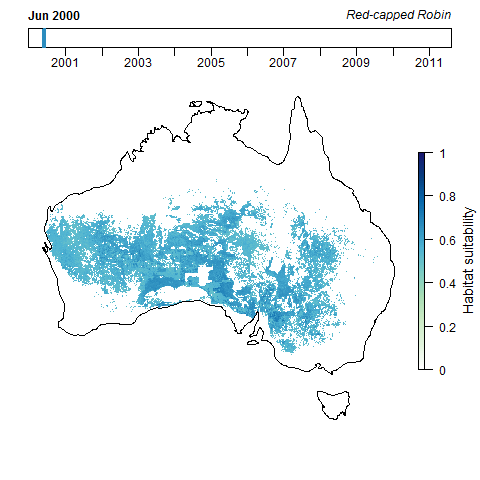

Supplement: Supplementary file 1 — Information on vegetation reclassification (Appendix S1) and range size metrics and model statistics (Appendix S2); plots of temporal dynamics in range size (Appendix S3); and animated maps of environmental suitability over time (Appendix S4) are available online for all 43 modeled species. The authors are solely responsible for the content and functionality of these materials. Queries (other than absence of the material) should be directed to the corresponding author. [file cobi0029-0865-sd1.zip › Supplementary-S4-Animations/Petroica_goodenovii.gif]

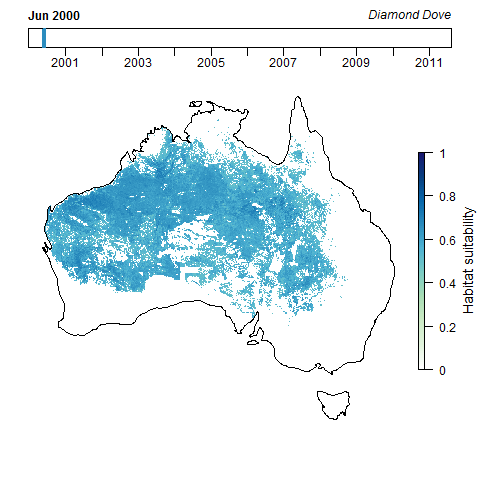

Supplement: Supplementary file 1 — Information on vegetation reclassification (Appendix S1) and range size metrics and model statistics (Appendix S2); plots of temporal dynamics in range size (Appendix S3); and animated maps of environmental suitability over time (Appendix S4) are available online for all 43 modeled species. The authors are solely responsible for the content and functionality of these materials. Queries (other than absence of the material) should be directed to the corresponding author. [file cobi0029-0865-sd1.zip › Supplementary-S4-Animations/Geopelia_cuneata.gif]

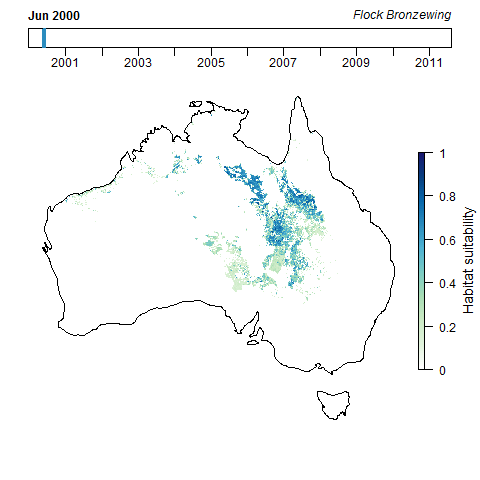

Supplement: Supplementary file 1 — Information on vegetation reclassification (Appendix S1) and range size metrics and model statistics (Appendix S2); plots of temporal dynamics in range size (Appendix S3); and animated maps of environmental suitability over time (Appendix S4) are available online for all 43 modeled species. The authors are solely responsible for the content and functionality of these materials. Queries (other than absence of the material) should be directed to the corresponding author. [file cobi0029-0865-sd1.zip › Supplementary-S4-Animations/Phaps_histrionica.gif]

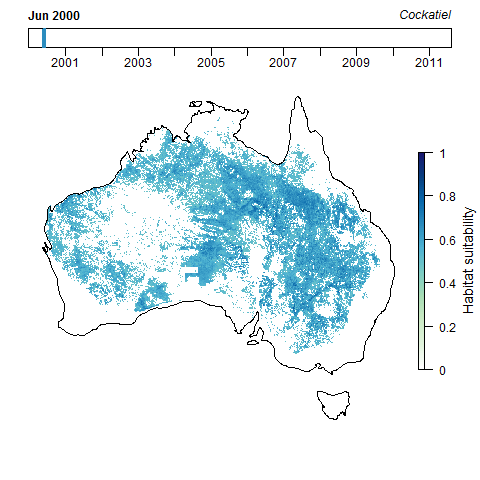

Supplement: Supplementary file 1 — Information on vegetation reclassification (Appendix S1) and range size metrics and model statistics (Appendix S2); plots of temporal dynamics in range size (Appendix S3); and animated maps of environmental suitability over time (Appendix S4) are available online for all 43 modeled species. The authors are solely responsible for the content and functionality of these materials. Queries (other than absence of the material) should be directed to the corresponding author. [file cobi0029-0865-sd1.zip › Supplementary-S4-Animations/Nymphicus_hollandicus.gif]

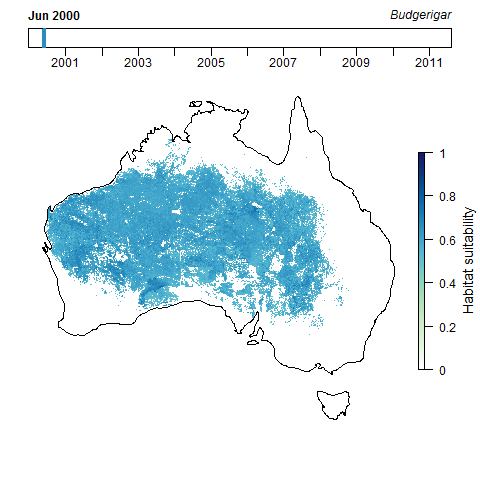

Supplement: Supplementary file 1 — Information on vegetation reclassification (Appendix S1) and range size metrics and model statistics (Appendix S2); plots of temporal dynamics in range size (Appendix S3); and animated maps of environmental suitability over time (Appendix S4) are available online for all 43 modeled species. The authors are solely responsible for the content and functionality of these materials. Queries (other than absence of the material) should be directed to the corresponding author. [file cobi0029-0865-sd1.zip › Supplementary-S4-Animations/Melopsittacus_undulatus.gif]

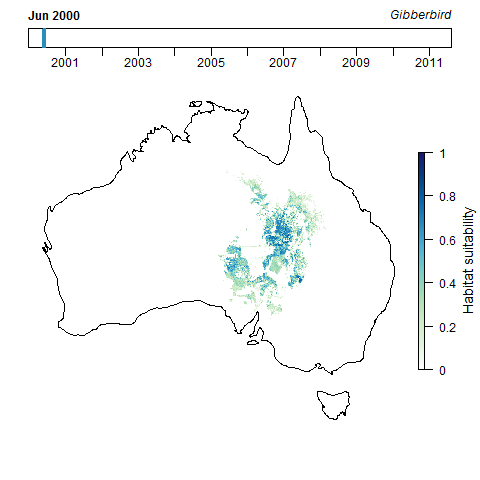

Supplement: Supplementary file 1 — Information on vegetation reclassification (Appendix S1) and range size metrics and model statistics (Appendix S2); plots of temporal dynamics in range size (Appendix S3); and animated maps of environmental suitability over time (Appendix S4) are available online for all 43 modeled species. The authors are solely responsible for the content and functionality of these materials. Queries (other than absence of the material) should be directed to the corresponding author. [file cobi0029-0865-sd1.zip › Supplementary-S4-Animations/Ashbyia_lovensis.gif]

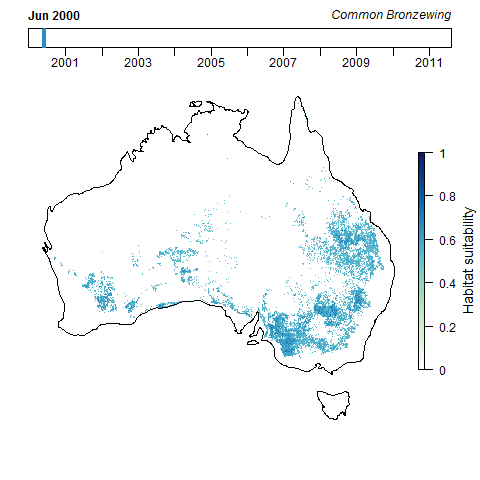

Supplement: Supplementary file 1 — Information on vegetation reclassification (Appendix S1) and range size metrics and model statistics (Appendix S2); plots of temporal dynamics in range size (Appendix S3); and animated maps of environmental suitability over time (Appendix S4) are available online for all 43 modeled species. The authors are solely responsible for the content and functionality of these materials. Queries (other than absence of the material) should be directed to the corresponding author. [file cobi0029-0865-sd1.zip › Supplementary-S4-Animations/Phaps_chalcoptera.gif]

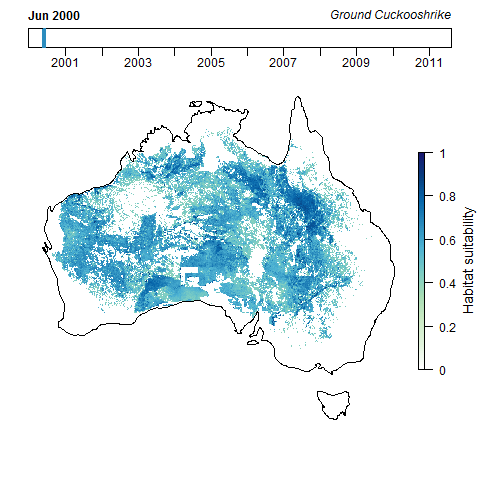

Supplement: Supplementary file 1 — Information on vegetation reclassification (Appendix S1) and range size metrics and model statistics (Appendix S2); plots of temporal dynamics in range size (Appendix S3); and animated maps of environmental suitability over time (Appendix S4) are available online for all 43 modeled species. The authors are solely responsible for the content and functionality of these materials. Queries (other than absence of the material) should be directed to the corresponding author. [file cobi0029-0865-sd1.zip › Supplementary-S4-Animations/Coracina_maxima.gif]

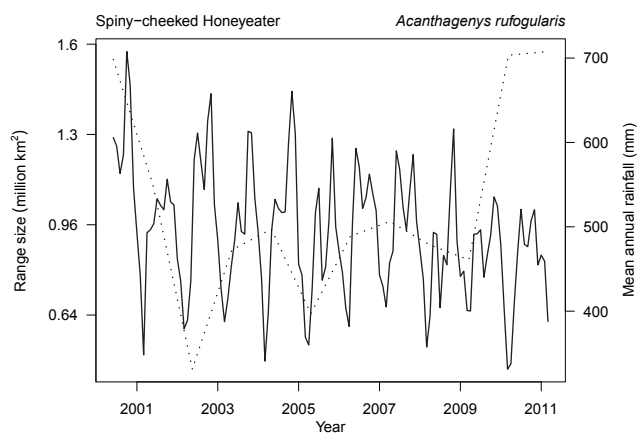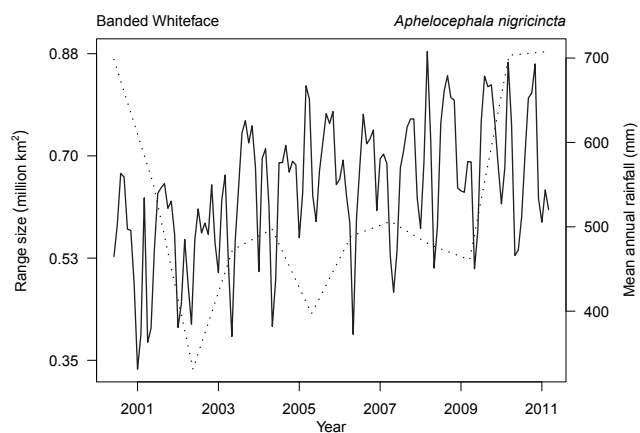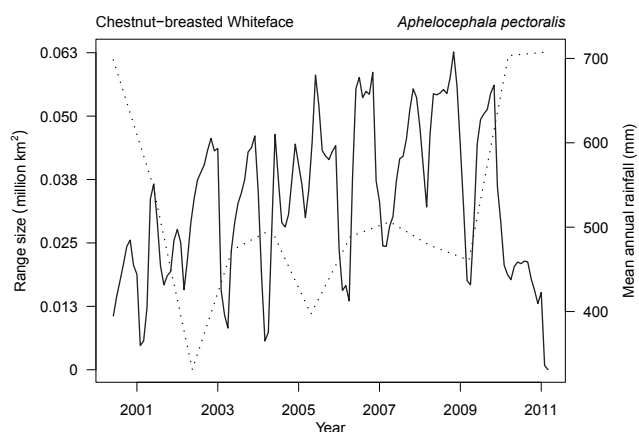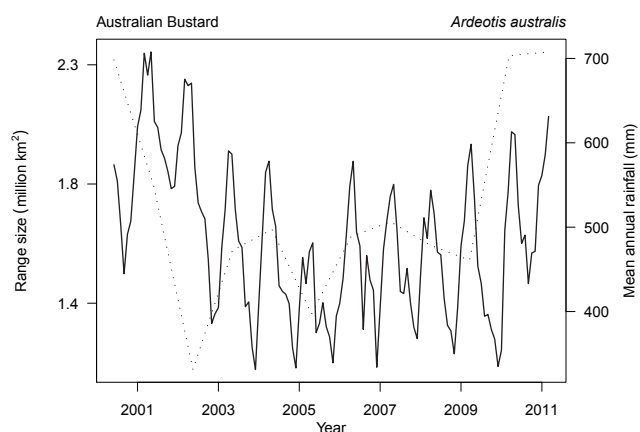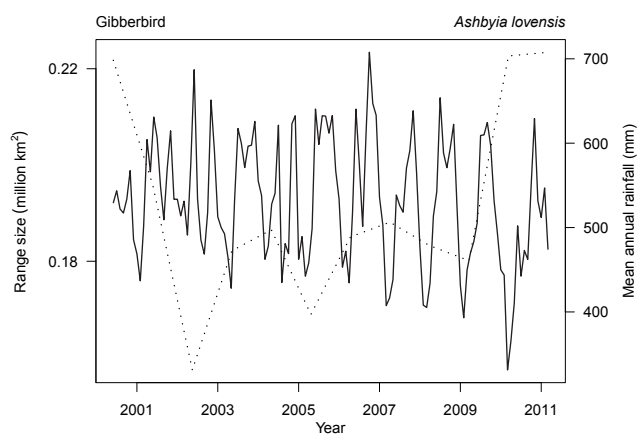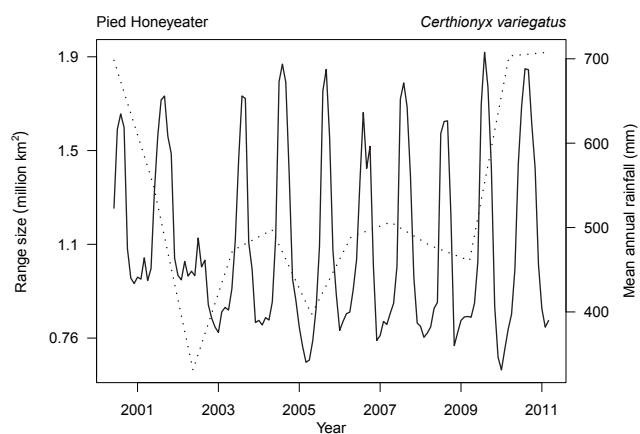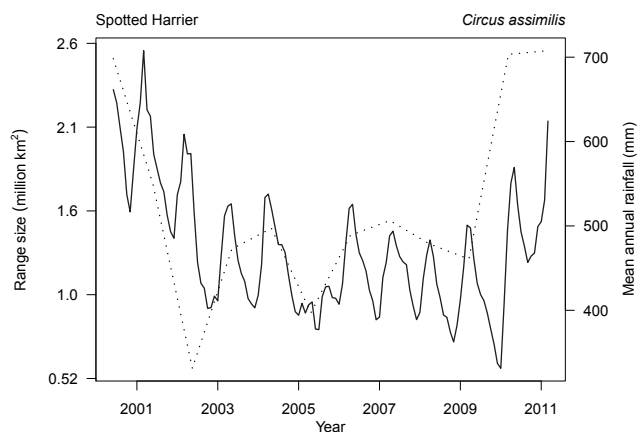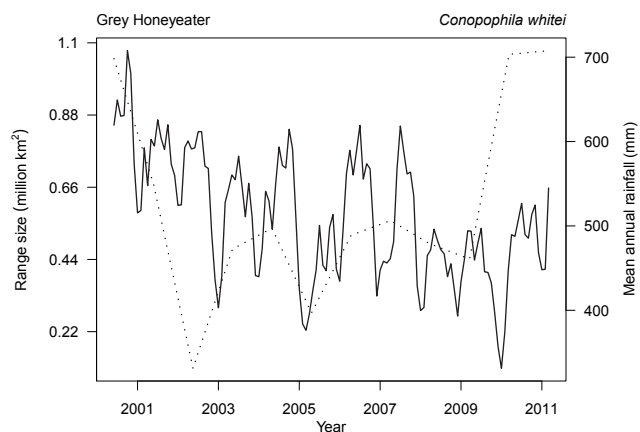

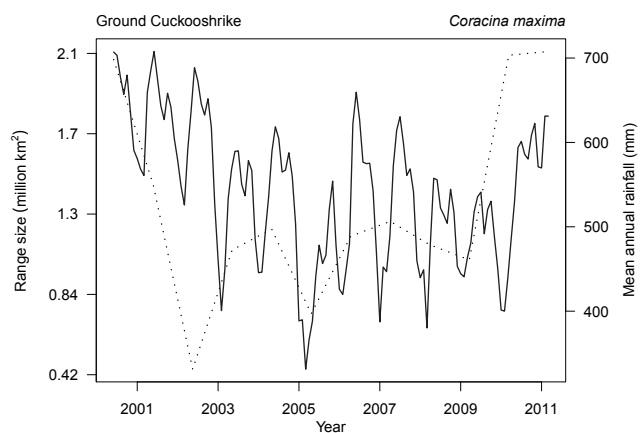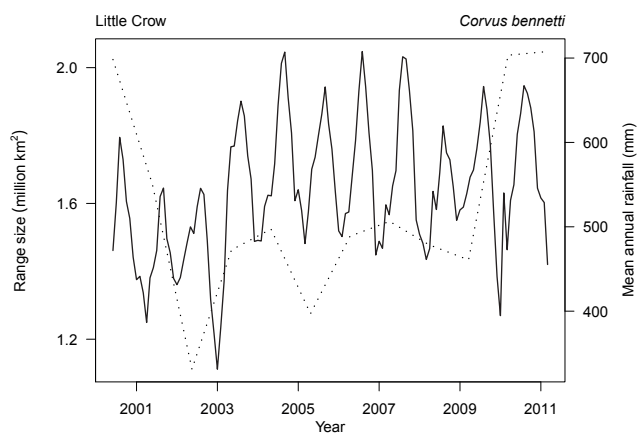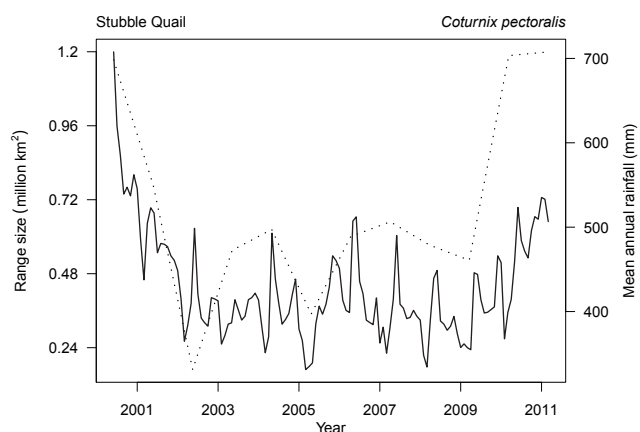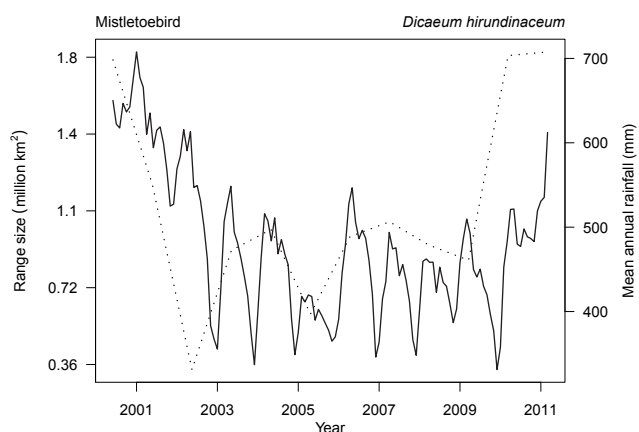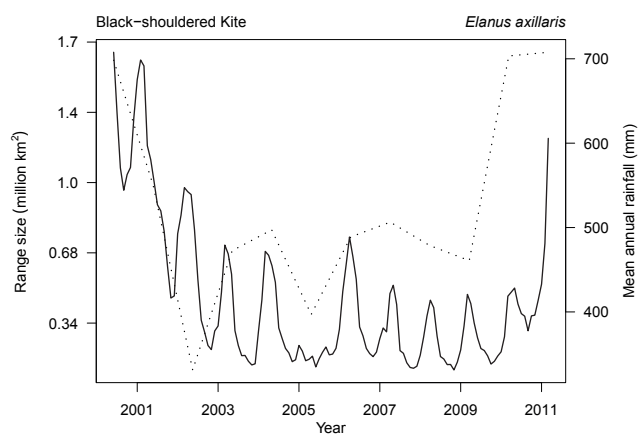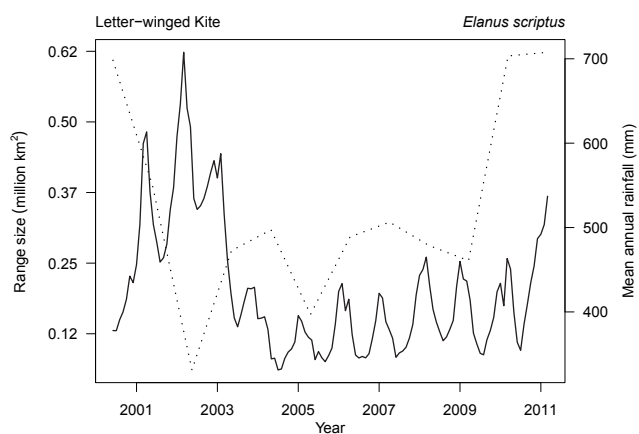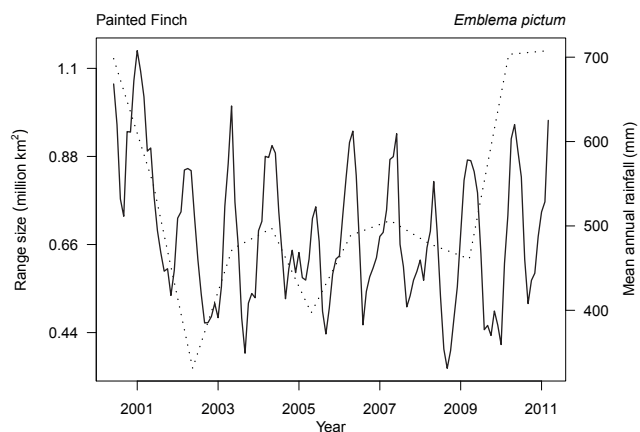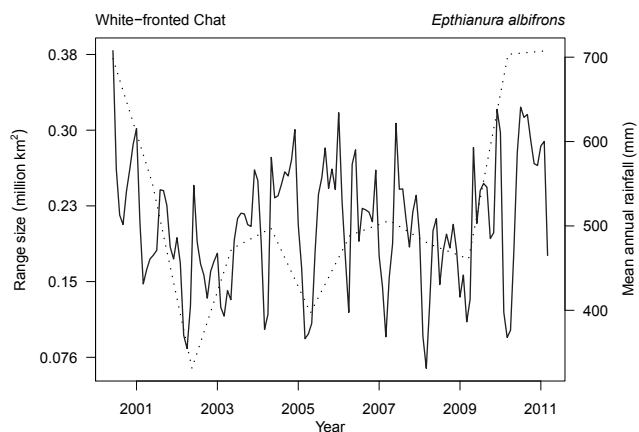

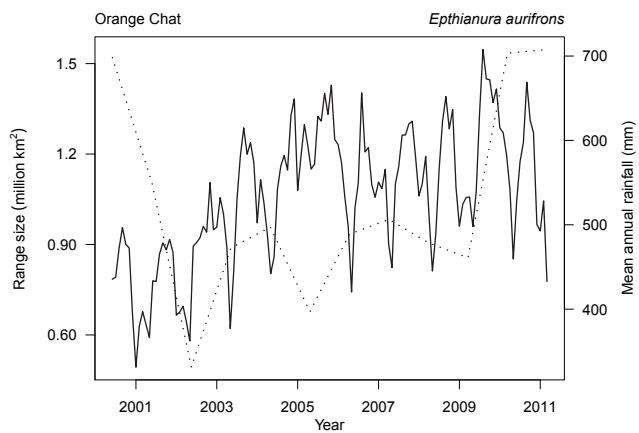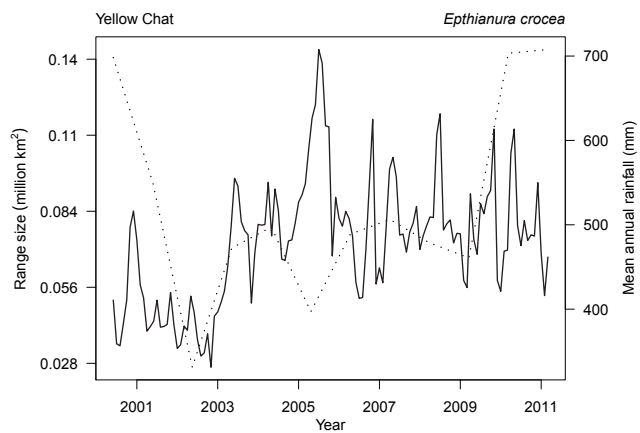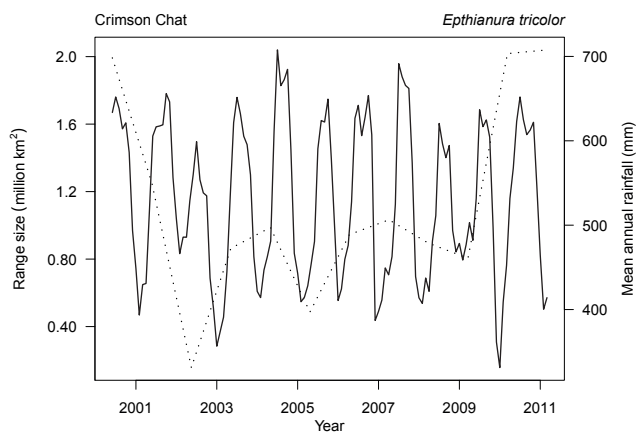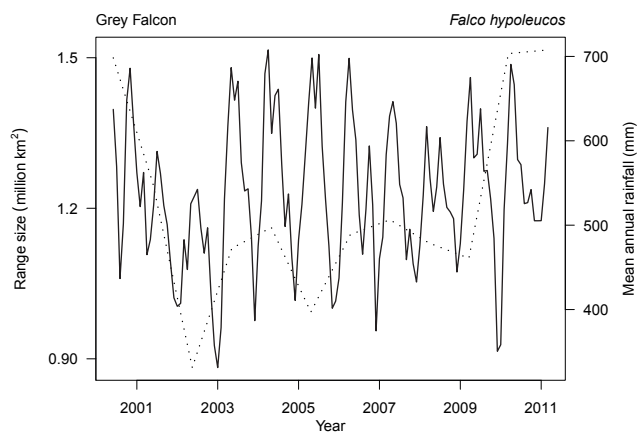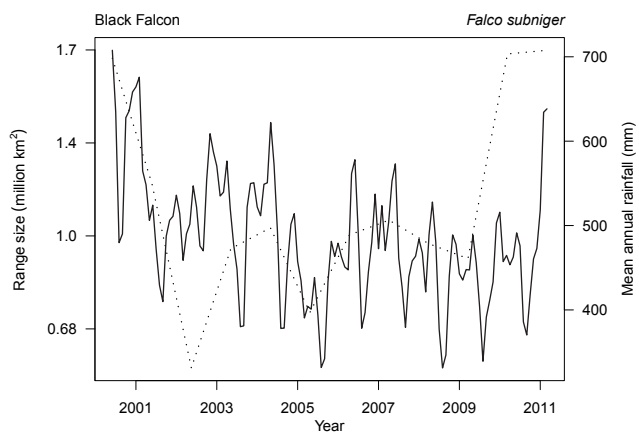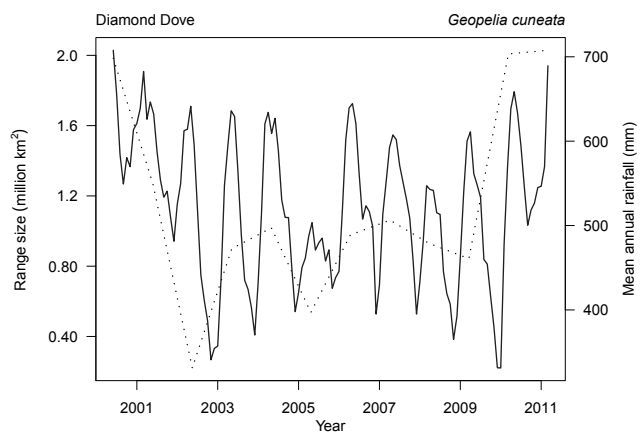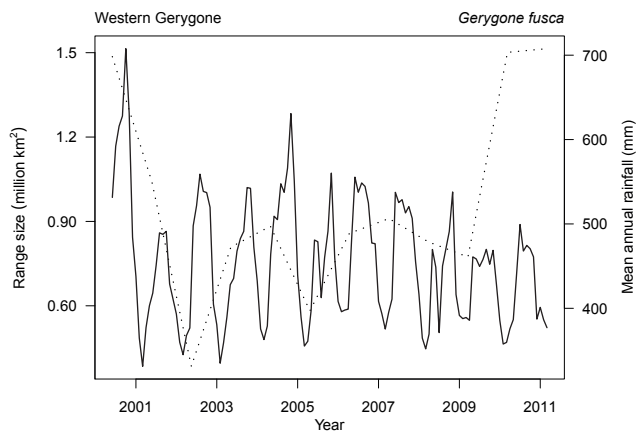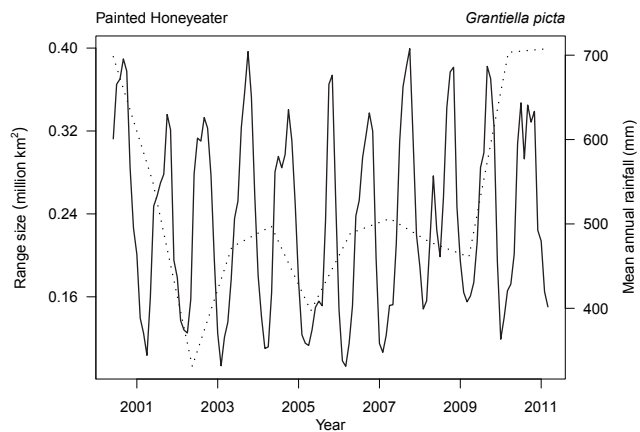

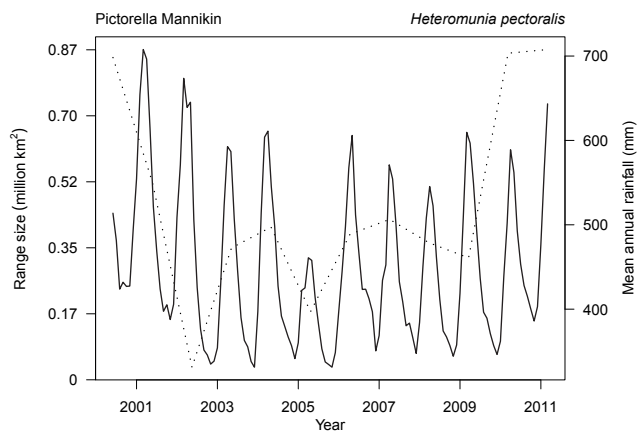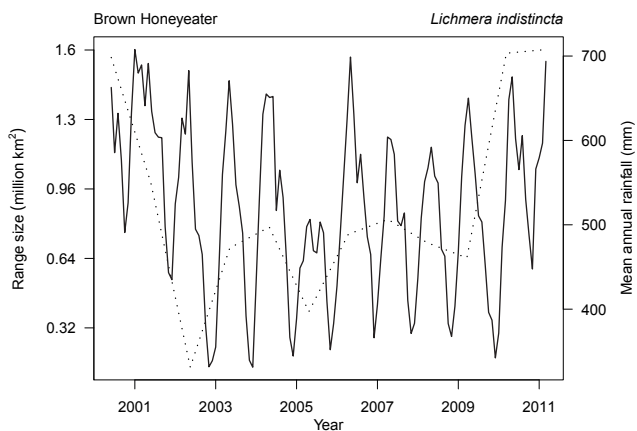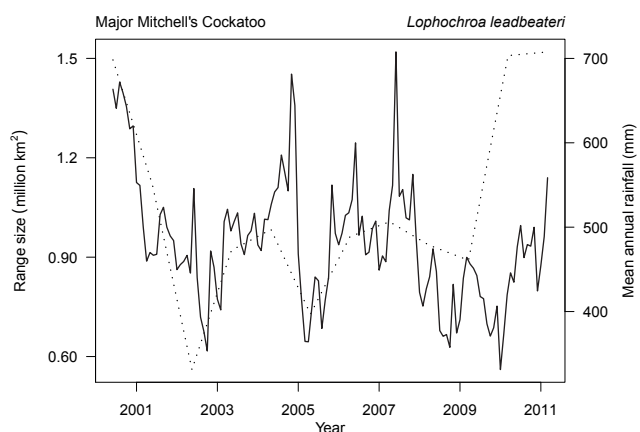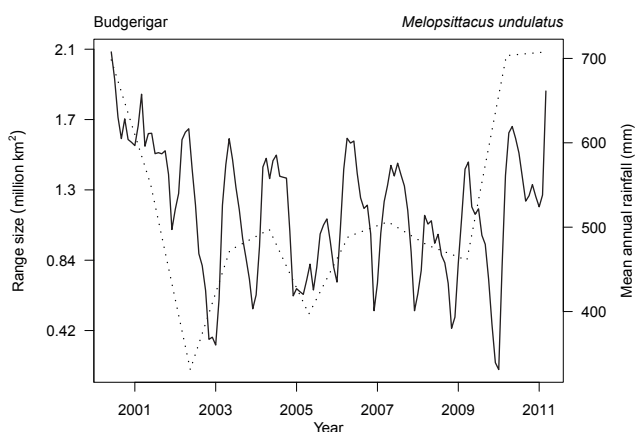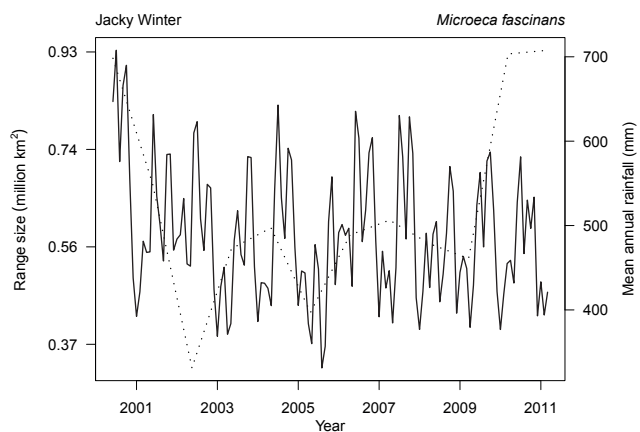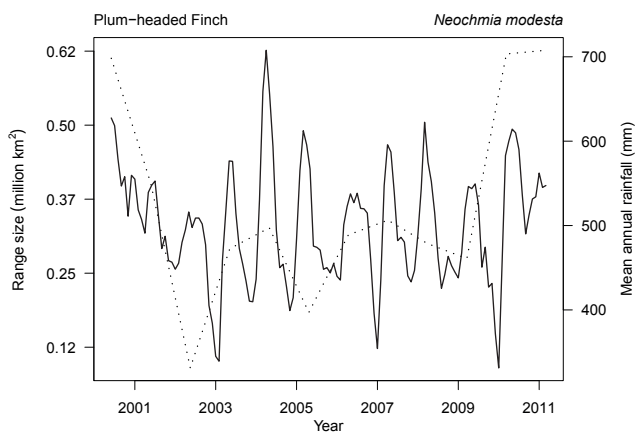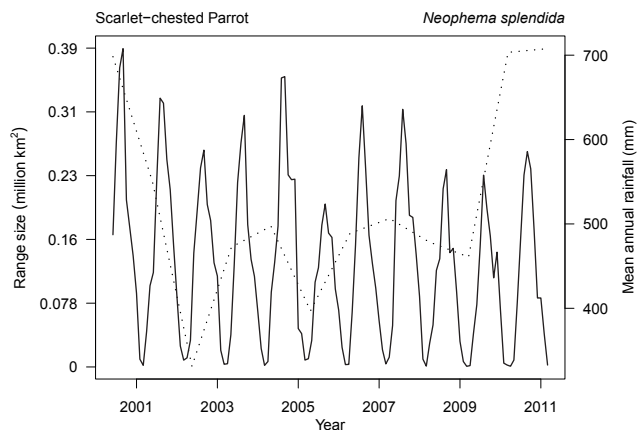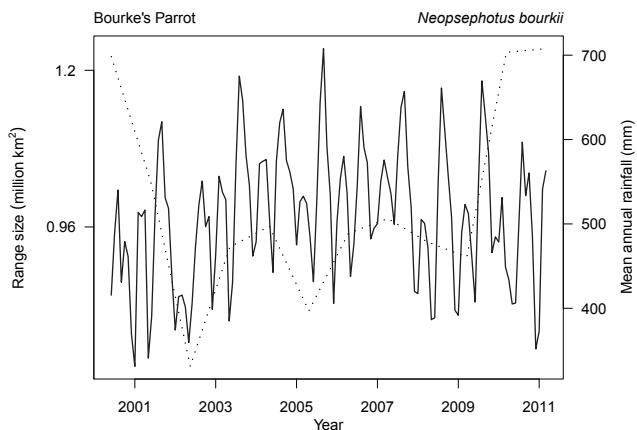

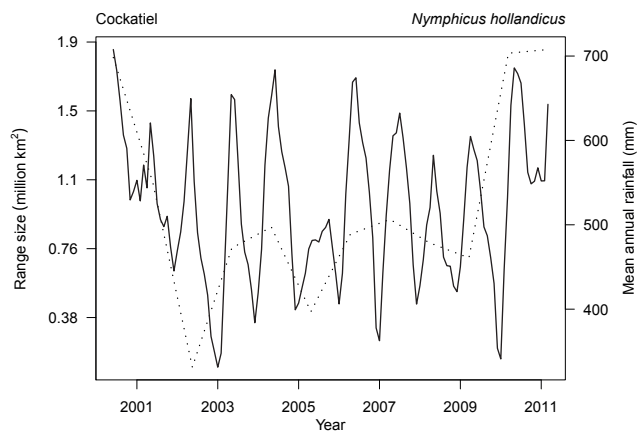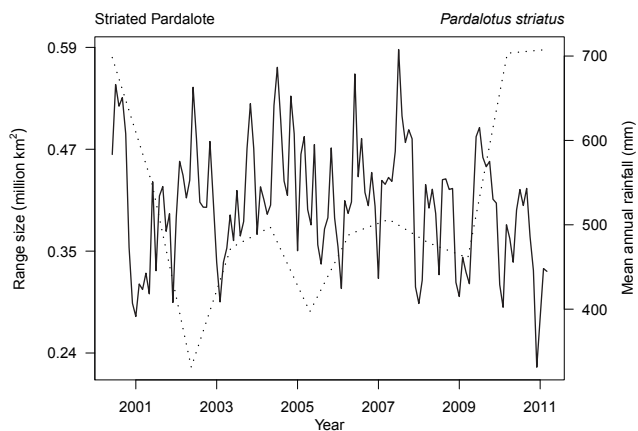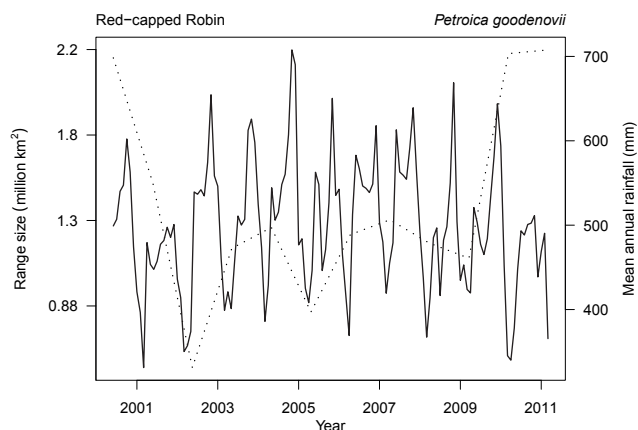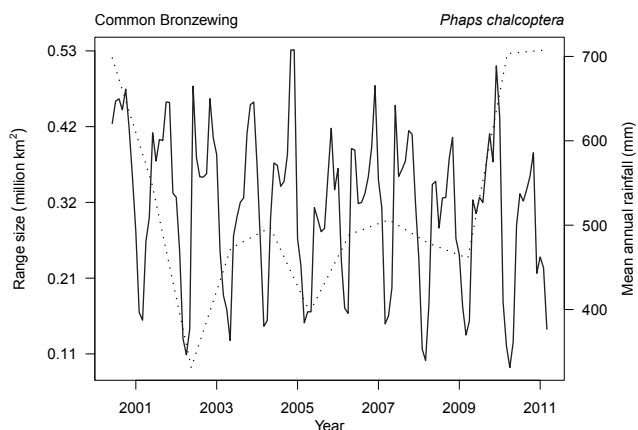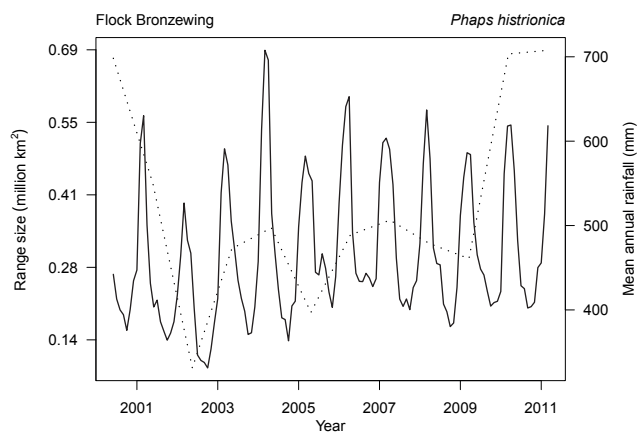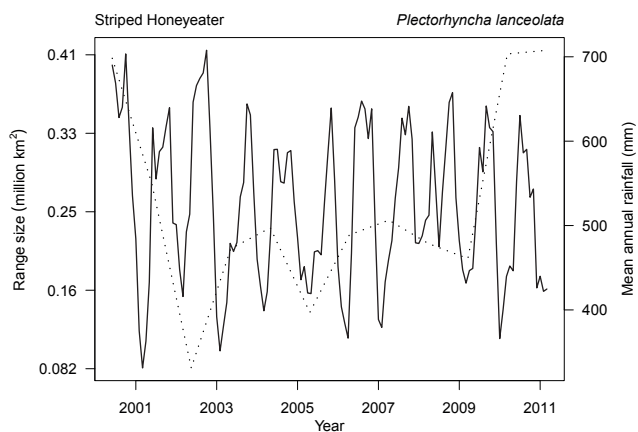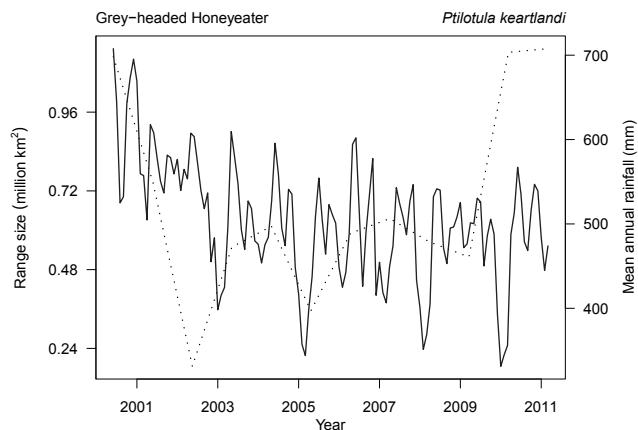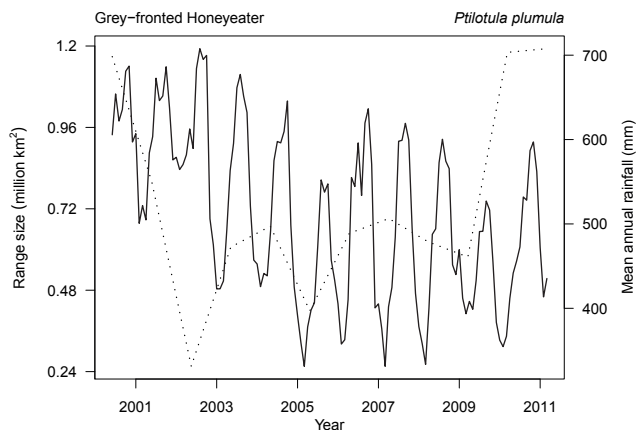

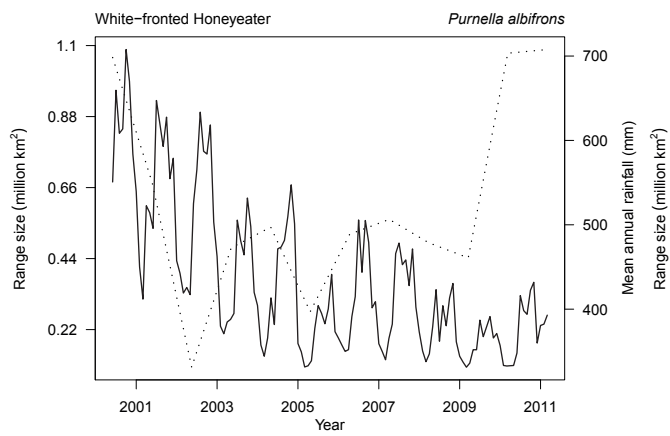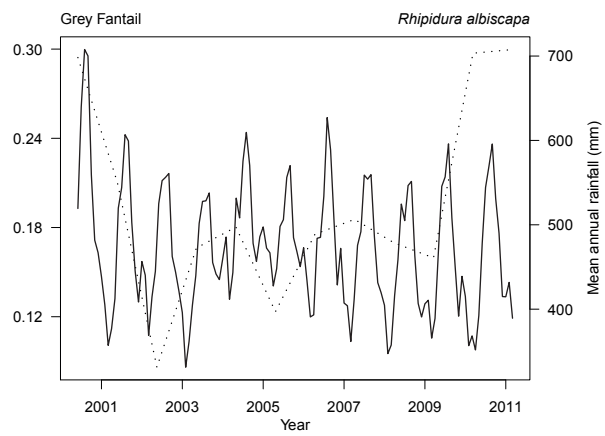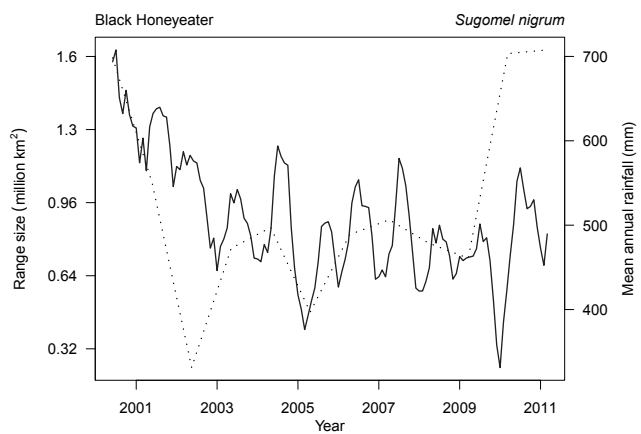

Supplement: Supplementary file 1 — Information on vegetation reclassification (Appendix S1) and range size metrics and model statistics (Appendix S2); plots of temporal dynamics in range size (Appendix S3); and animated maps of environmental suitability over time (Appendix S4) are available online for all 43 modeled species. The authors are solely responsible for the content and functionality of these materials. Queries (other than absence of the material) should be directed to the corresponding author. [file cobi0029-0865-sd1.zip › cobi12440-sup-0001-text-S3.pdf]
